# NR5A2 Is One of 12 Transcription Factors Predicting Prognosis in HNSCC and Regulates Cancer Cell Proliferation in a p53-Dependent Manner

**DOI:** 10.3389/fonc.2021.691318

**Published:** 2021-07-01

**Authors:** Kun Zhang, Ming Xiao, Xin Jin, Hongyan Jiang

**Affiliations:** ^1^ Department of Otorhinolaryngology, Union Hospital, Tongji Medical College, Huazhong University of Science and Technology, Wuhan, China; ^2^ Department of Urology, The Second Xiangya Hospital, Central South University, Changsha, China

**Keywords:** head and neck squamous cell carcinoma, transcription factors, NR5A2, p53, cell proliferation

## Abstract

Head and neck squamous cell carcinoma (HNSCC) rank seventh among the most common type of malignant tumor worldwide. Various evidences suggest that transcriptional factors (TFs) play a critical role in modulating cancer progression. However, the prognostic value of TFs in HNSCC remains unclear. Here, we identified a risk model based on a 12-TF signature to predict recurrence-free survival (RFS) in patients with HNSCC. We further analyzed the ability of the 12-TF to predict the disease-free survival time and overall survival time in HNSCC, and found that only NR5A2 down-regulation was strongly associated with shortened overall survival and disease-free survival time in HNSCC. Moreover, we systemically studied the role of NR5A2 in HNSCC and found that NR5A2 regulated HNSCC cell growth in a TP53 status-dependent manner. In p53 proficient cells, NR5A2 knockdown increased the expression of *TP53* and activated the p53 pathway to enhance cancer cells proliferation. In contrast, NR5A2 silencing suppressed the growth of HNSCC cells with p53 loss/deletion by inhibiting the glycolysis process. Therefore, our results suggested that NR5A2 may serve as a promising therapeutic target in HNSCC harboring loss-of-function *TP53* mutations.

## Introduction

Head and neck squamous cell carcinoma (HNSCC) accounts for more than 95% of all head and neck malignant tumor. To date, HNSCC ranks seventh among the most common type of malignant tumor worldwide (1). Despite progress in treatment methods for HNSCC, the five-year survival rate of HNSCC patients remains less than 50% (2–4). Thus, the identification of new prognostic factors and therapeutic targets for HNSCC is an urgent need.

Various transcription factors (TFs) have been identified as critical players in malignant transformation and tumor progression (5). Li et al. reported that HOX family TFs are important factors promoting carcinogenesis (6). Similarly, Carter et al. have demonstrated that the prognostic value of the TFs WT1 and p53 in ovarian cancer (7). However, inconsistencies exist among studies and types of cancer regarding the prognostic role of these TFs. Limited sample availability and differences among cancer types and data processing strategies are significant factors contributing to these inconsistencies.

In this study, a systematic and comprehensive analysis of gene expression data and clinical information of HNSCC patients was performed to identify TFs acting as reproducible and robust biomarkers for HNSCC. We established a 12-TF signature predicting recurrence-free survival (RFS) in patients with HNSCC. Importantly, we found that nuclear receptor subfamily 5 group A member 2 (NR5A2) downregulation was strongly associated with poor HNSCC patient survival. Intriguingly, the tumor-suppressive ability of NR5A2 was affected by the p53 expression level. These findings strongly support the prognostic potential of the 12-TF signature and indicate that NR5A2 represent a new therapeutic target in HNSCC.

## Materials and Methods

### Data Acquisition and Processing

Genes expression data and clinical information of HNSCC patients were acquired from the TCGA database using the “TCGA biolinks” package (8). Data from 528 HNSCC patients and 24991 genes were acquired. Patients lacking prognostic data or non-transcription factor genes for survival analysis were exclude from further analysis. TFs were downloaded from TRRUST database (https://www.grnpedia.org/trrust/downloadnetwork.php) (9). Subsequent analyses include data from 499 HNSCC patients and 795 TFs. Data from 270 HNSCC patients obtained from GEO database (GSE65858) were used as an external validation set.

### Identification of Prognostic TFs and Receiver Operating Characteristic (ROC) Analysis

Univariate Cox regression analysis was conducted to screen TFs predicting RFS in HNSCC patients. Then, these screened TFs went through LASSO Cox regression analysis to further select TFs for predicting RFS of HNSCC. A prognostic TFs signature related to the RFS of HNSCC patients was constructed by the multiple Cox regression; a 12-TF prognostic signature was identified based on a linear combination of the regression coefficient, which was derived from the multiple Cox regression analysis. For each sample, the estimated risk score was obtained from the Cox regression model containing the 12 TFs. Using the average risk scores as the cutoff value, we stratified all the eligible patients into high- and low-risk cohorts. To assess the performance of the 12-TF signature in predicting RFS in patients with HNSCC, we performed time-dependent ROC curve analyses using the “survival ROC” R package. The model with a higher area under the curve (AUC) value was more favorable for the hazard prediction. The differences in RFS between the high- and low-risk groups were evaluated *via* the Kaplan–Meier method, followed by a log-rank test based on the survival the R package “survival”. R (version 3.6.1) was used to plot all ROC and Kaplan–Meier curves. The prognostic performance was internally cross validated by 3-fold approach in TCGA-HNSCC dataset and externally validated in GSE65858 dataset using the coefficients derived from the TCGA-HNSCC dataset.

### Cell Culture and Chemicals

The FaDu (#HTB-43), SCC-4 (#CRL-1624), SCC-15 (#CRL-1623)and SCC-9 (#CRL-1629) cell lines were purchased from ATCC. FaDu, SCC-4, SCC-15 and SCC-9 cells were maintained in Dulbecco’s Modified Eagle Medium (#A4192101, Thermo Fisher) with 10% fetal bovine serum (#10099141, Thermo Fisher) in a 5% CO2 incubator at 37°C. GAPDH antibody (#ab8245, working dilution 1:3000) was purchased from Abcam; NR5A2 antibody (#22460-1-AP, working dilution 1:800) and p53 antibody (#60283-2-Ig, working dilution 1:1000) were obtained from Proteintech. siRNAs were purchased from RIBOBIO CO.,LTD. (GUANGZHOU, China). siRNAs were transfected into the cells by using the Lipofectamine 2000 (Invitrogen, USA).

### Western Blotting Analysis

The lysis buffer supplemented with 1% protease and phosphatase inhibitors was used to lyse the collected cells on ice for 15 min. The proteins were obtained bycentrifuged cell lysates at 12,000 g at 4°C for 15 min, and the supernatants were send for measure the protein concentration by using the Protein quantification kit (#P0012S, Beyotime). Each well of the SDS-PAGE gel is loaded with the same amount of total protein and proteins were transferred onto the PVDF membrane. Then, 5% nonfat milk was using to block the membranes for 60 min at room temperature. Subsequently, the membranes were incubated with the primary antibody and secondary antibody at 4°C for 10 h and at room temperature for 1 h, respectively. At last, ECL detection reagents (#WP20005, Thermo Fisher) were added on the surface of the membranes to expose the protein bands under X-ray.

### shRNA Interference

The shRNAs were synthesized by Sigma-Aldrich. ShRNA plasmids together with pVSV-G and pEXQV were transfected into the 293T cells with Lipo2000™ Transfection Reagent (#c0562, Beyotime, China). After 1 d, the medium was changed with fresh Dulbecco’s Modified Eagle Medium plus sodium pyruvate (1 mM). 2 days later, the medium containing virus of shRNA was collected and co-cultured with HNSCC cells for other 24 h. Then, 1 μg/ml puromycin were added to the medium to select the infected HNSCC cells. The shRNA sequence is shown in **Table S3**.

### Tissue Microarray and Immunohistochemistry (IHC)

The tissue microarray slides were purchased from Avilabio (Xi’an, China) (DC-Hea11012). The tissue microarray specimens were immunostained with NR5A2 (Proteintech, #22460-1-AP, working dilution 1:800). Staining intensity was scored in a blinded fashion: 1 = weak staining at 100× magnification but little or no staining at 40× magnification; 2 = medium staining at 40× magnification; 3 = strong staining at 40× magnification. The degree of immunostaining was reviewed and scored by two independent pathologists who were blinded to the clinical details. The scores were determined by the percentage of positive cells multiplied by the staining intensity.

### Chromatin Immunoprecipitation (ChIP) and ChIP-qPCR

For ChIP assay, Chromatin Extraction Kit (Abcam, ab117152, USA) and ChIP Kit Magnetic - One Step (Abcam, ab156907, USA) was used to preform ChIP following the manufacture’s instruction. The detailed antibodies as follows: NR5A2 (Proteintech; 22460-1-AP; 1:100). TB Green™ Fast qPCR Mix kit (Cat. No. RR430A) purchased from Takara Bio Inc. (Shiga, Japan) were used to perform qPCR analysis. The sequences of primers were provided in **Table S2**.

### Statistical Analysis

GraphPad Prism8 software (GradPad Software, Inc) was used for all statistical analyses. The Paired t-test, Student’s t-test, and one or two-way ANOVA were applied to assess the statistical significance. Only a P value less than 0.05 is considered significant. All values are indicated as mean ± SD.

## Results

### Identification of TFs Predicting RFS in Patients With HNSCC

**Figure 1:**
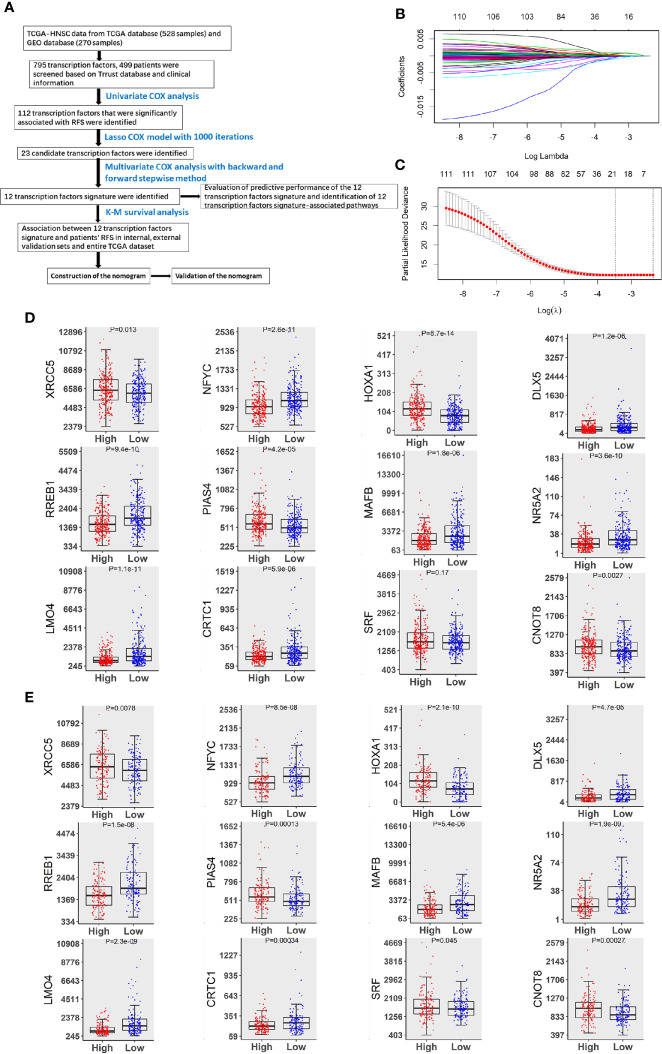
Identification of TFs predicting RFS in patients with HNSCC. **(A)** The flow chart of how to identify TFs associated with RFS in HNSCC. **(B)** 10-fold cross-validation for tuning parameter selection in the LASSO model by minimum criteria (the 1-SE criteria). **(C)** LASSO coefficient profiles of the 112 TFs genes. A coefficient profile plot was conducted against log (lambda) sequence. Vertical line was drawn at the value selected with 10-fold cross-validation, in which optimal lambda led to 23 non-zero coefficients. **(D)** Boxplots of the 12 TFs expression values against risk group in the TCGA dataset. “High” and “Low” referred to the high-risk and low-risk clusters, respectively. The differences between the 2 clusters were evaluated by Mann-Whitney U test, and P values were observed in the graphs. **(E)** Boxplots of 12 TFs expression values against risk cluster in the GEO dataset.

In this study, we analyzed data from 499 patients who were diagnosed with HNSCC. The detailed clinicopathological features of this study cohort are shown in **Table S4**, and the study design is summarized in **Figure 1A**. By performing univariate Cox regression analyses, we identified 112 TFs that were related to HNSCC patients’ RFS (P < 0.05). Twenty-three TFs were further selected by LASSO Cox regression with the optimal lambda value (**Figures 1B, C**). Of these 23 TFs, 12 TFs (XRCC5, NFYC, HOXA1, DLX5, RREB1, PIAS4, MAFB, NR5A2, LMO4, CRTC1, SRF and CNOT8) were significantly associated with RFS in patients with HNSCC according to the findings of multiple Cox analyses (*P* < 0.05). Notably, high levels of XRCC5, HOXA1, PIAS4, SRF and CNOT8 were correlated with increasing the risk of recurrence. On the other hand, high levels of NFYC, DLX5, RREB1, MAFB, NR5A2, LMO4, and CRTC1 were associated with a decreased risk of recurrence (**Figures 1D, E**). These findings suggest that the 12-TF signature has a prognostic value in HNSCC.

### Prognostic Performance of the 12-TF Signature in HNSCC

**Figure 2:**
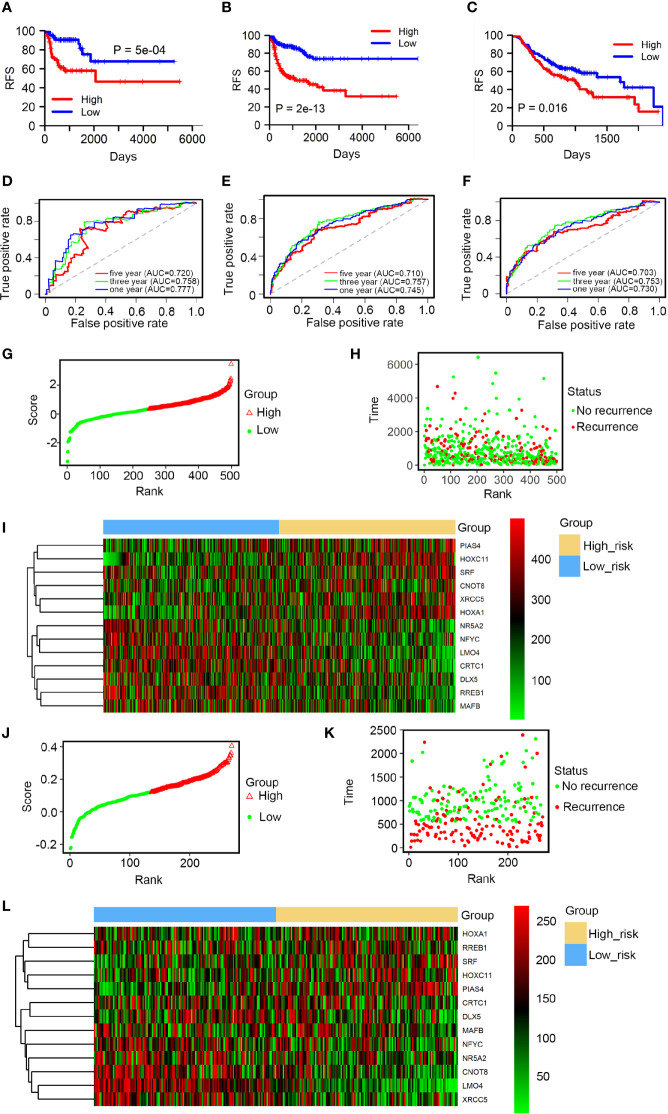
Prognostic performance of the 12-TF signature in HNSCC. **(A–C)** Kaplan-Meier and ROC analysis of patients with HNSCC in entire TCGA set, the internal validation and external validation, respectively. **(D–F)** Kaplan-Meier analysis with two-sided log-rank test was conducted to evaluate the differences in RFS between the low-risk and high-risk cases. 1-, 3-, 5-year ROC curves of the 12-TF signature were applied to measure the value in predicting HNSCC patients’ RFS in entire TCGA set, the internal validation and external validation, respectively. **(G–I)** TF risk score analysis of 499 HNSCC samples in TCGA database. TF risk score distribution against the rank of risk score **(G)**. Average risk score was set as the cut-off point. Recurrence free survival status of HNSCC patients **(H)**. Heatmap of 12 TFs expression profiles of HNSCC patients **(I)**. **(J–L)** TF risk score analysis of 270 HNSCC samples in the GEO dataset. TF risk score distribution against the rank of risk score **(J)**. Average risk score was set as the cut-off point. Recurrence free survival status of HNSCC patients **(K)**. Heatmap of 12 TFs expression profiles of HNSCC patients **(L)**.

Using the average risk score as the cutoff value, HNSCC patients were split into high and low-risk groups. The differences in RFS between the 2 groups were assessed by Kaplan-Meier survival analysis. Patients in the low-risk group showed a longer RFS than in the high-risk group (**Figure 2A**). These findings were confirmed by internal validation (**Figure 2B**) and independent external validation (**Figure 2C**). The prognostic performance of the 12-TF signature was evaluated using ROC curve. The area under the AUC curve values at 1, 3, and 5 years were 0.777, 0.758, and 0.720, respectively (**Figure 2D**). The high prognostic performance of the signature was further confirmed by internal validation (AUC: 0.745, 0.757, and 0.710; **Figure 2E**) and external validation (AUC: 0.730, 0.753, and 0.703; **Figure 2F**). These results further support the potential use of the 12-TF signature to predict recurrence in patients with HNSCC. Moreover, patients were ranked based on risk scores (**Figures 2G, J**), and their survival statuses were plotted (**Figures 2H, K**). A heatmap of the expression levels of the 12 TFs grouped based on the risk score is shown in **Figures 2I, L**. This analysis confirmed the low-risk cohort had a lower recurrence rate than the high-risk cohort.

### Biological Functions and Clinical Applications of the 12 TFs

**Figure 3:**
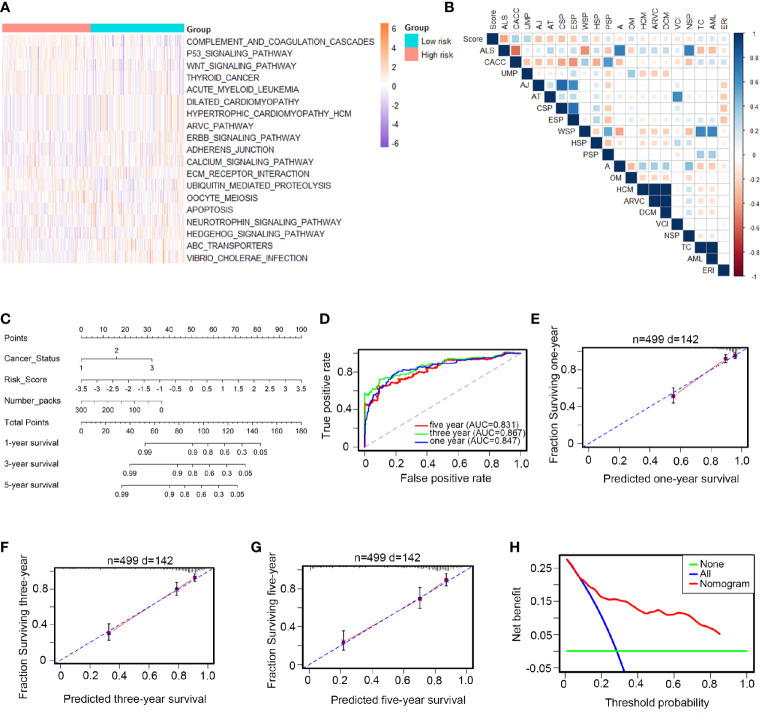
Biological functions and clinical applications of the 12 TFs. **(A, B)** Pathway profiles in the light of TCGA dataset. Rows referred to pathways, and columns stood for patients. Each grid stood for a score of pathway activity calculated by single-sample GSEA. The ssGSEA score was further rescaled by min-max normalization method. The upper horizontal bar marked the information involved in every case which contained risk cluster and risk score (from low to high). In order to fit the picture size, the pathway labels in **(B)** were handled as abbreviations of the corresponding pathway names in **(A)**. **(C)** The nomogram was performed in the entire TCGA database, with cancer status as well as number packs. **(D)** 1-, 3-, 5-year ROC curves for the TFs-based nomogram. **(E–G)** Represented the 1-, 3-, 5-year nomogram calibration curves, respectively. The closer the dotted line fit to the perfect line, the better the predictive performance of the nomogram was. **(H)** The decision curve analysis (DCA) for the nomogram. The net benefit was plotted versus the threshold probability. The red line was the nomogram. The blue line was the treat-all and the green was the treat-none.

HNSCC patients were divided into low- and high-risk groups according to the average risk score as the cutoff value. The top 20 core transcriptional networks associated with the risk score are shown in **Figures 3A, B**. Univariate and multiple and Cox analyses were performed to assess the potential use of the 12-TF signature as an independent prognostic marker in HNSCC; the hazard ratios (HRs) are shown in **Table S5**. Notably, the levels of the 12-TF signature were significantly associated with RFS in patients with HNSCC (HR, 2.22; 95% CI, 1.78-2.77; *P <*0.001) independently of other clinicopathological variables. Additionally, we developed a nomogram based on the risk score, cancer status, and number packs (**Figure 3C**). The AUC values (0.847, 0.867, 0.831; **Figure 3D**) and calibration plots indicated the high prognostic performance of the signature (**Figures 3E–G**). DCA demonstrated that the net benefit associated with the use of the nomogram for the three-year recurrence (**Figure 3H**). These results indicate that our nomogram is a robust predictor of RFS in patients with HNSCC.

### Role of NR5A2 in Cell Proliferation in Different HNSCC Cell Lines

**Figure 4:**
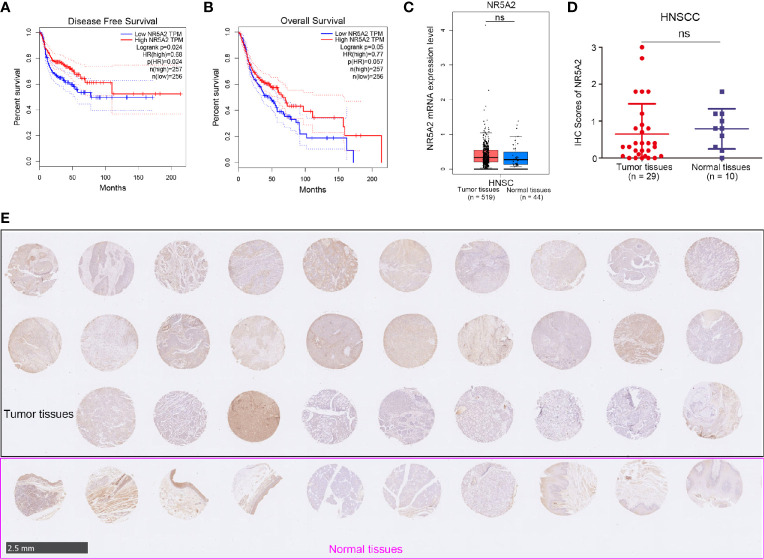
The clinical-pathological feature of NR5A2 in HNSCC. **(A, B)** The disease-free survival **(A)** and overall survival **(B)** of NR5A2 in HNSCC analyzed by the GEPIA web tool, P values as indicated. **(C)** The mRNA expression level of NR5A2 analyzed by the GEPIA web tool, n.s., not significant. **(D, E)** The protein levels of NR5A2 from the tissue microarray (n = 10 normal tissues, n = 29 HNSCC tumor tissues) were determined by IHC analysis, n.s., not significant.

**Figure 5:**
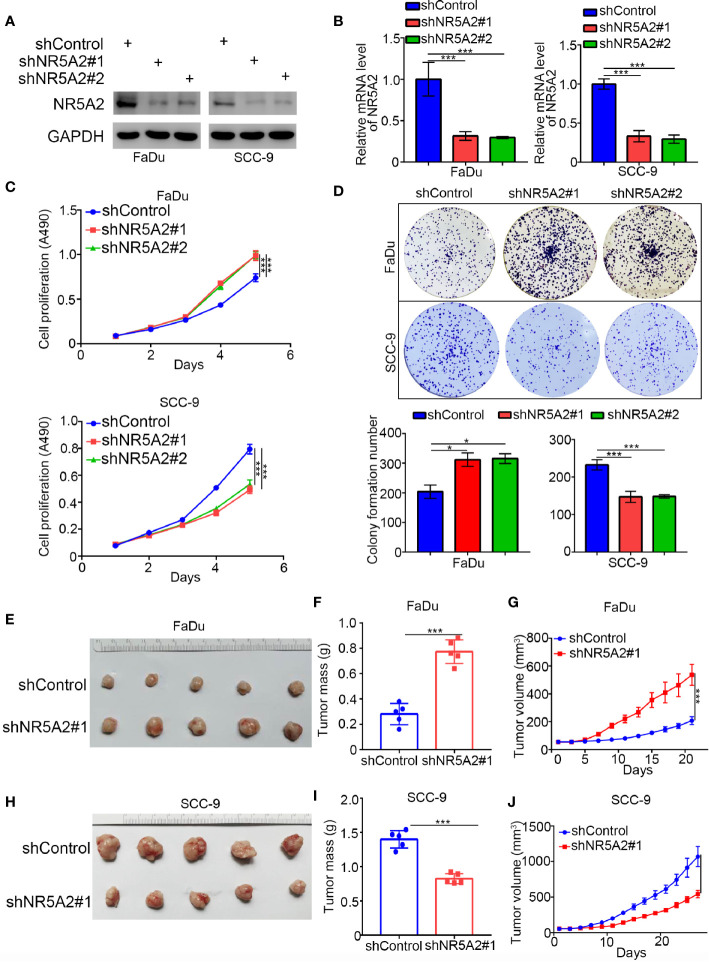
Role of NR5A2 in cell proliferation in different HNSCC cell lines. **(A, B)** Western blot analysis **(A)** and RT-qPCR **(B)** of NR5A2 expression in FaDu and SCC-9cells treated with indicated shRNAs. Data presented as the mean ± SD of three independent experiments. ***p < 0.001. **(C, D)** FaDu and SCC-9 cells infected with lentivirus vectors expressing control or NR5A2 specific shRNAs were harvested for MTS assay (n = 5), colony formation assay **(D)**. The photo was taken on the 14th day after 500 cells seeded, each bar represents the mean ± SD of three independent experiments. *P < 0.05; ***P < 0.001. **(E, G)** FaDu cells infected with shcontrol or shNR5A2 were injected subcutaneously into the right dorsal flank of nude mice. The tumor volume was measured every three days **(G)**. Then the tumors were harvested, photographed **(E)** and weighed **(F)**. The data are presented as the means ± SD (n = 5). ***P < 0.001. **(H, J)** SCC-9cells infected with shcontrol or shNR5A2 were injected subcutaneously into the right dorsal flank of nude mice. The tumor volume was measured every three days **(J)**. Then the tumors were harvested, photographed **(H)** and weighed **(I)**. The data are presented as the means ± SD (n = 5). ***P < 0.001.

Next, we used the GEPIA web tool to analyze the ability of individual TFs of the signature to predict disease-free survival (DFS) and overall survival (OS) in HNSCC (**Supplementary Figures 1A, B** and **Figures 4A, B**). Intriguingly, we showed that only NR5A2 downregulation was correlated with shortened DFS and OS in patients with HNSCC (**Figures 4A, B**). No significant association was observed between any of the other 11 TFs and DFS or OS in patients with HNSCC (**Supplementary Figures 1A, B**). However, *NR5A2* mRNA and protein levels did not differ significantly between HNSCC and non-malignant head and neck tissues (**Figures 4C–E**), suggesting that the role of NR5A2 in HNSCC may be two-faced. To further elucidate the role of NR5A2 in HNSCC, we silenced NR5A2 expression in FaDu, SCC-4, SCC-15 and SCC-9 cells using 2 different short hairpin RNAs (shRNAs) (**Figures 5A, B** and **Supplementary Figures 2A, B**). MTS assays revealed that NR5A2 silencing enhanced the growth of FaDu and SCC-4 cells (**Figure 5C** and **Supplementary Figures 2C, D**) but inhibited the growth of SCC-15 and SCC-9 cells (**Figure 5C** and **Supplementary Figures 2C, D**). Additionally, the colony formation assay showed that knockdown of NR5A2 enhanced FaDu cells proliferation and blocked SCC-9 cells growth (**Figure 5D**). Besides, we also evaluated the role of NR5A2 on the growth of mouse xenografts and found that NR5A2 silencing enhanced the growth of FaDu tumors (**Figures 5E–G**) but suppressed the growth of SCC-9 tumors (**Figures 5H–J**). These data suggest that the role of NR5A2 on HNSCC cell growth varies among cell lines.

### NR5A2 Regulates HNSCC Cell Growth in a p53-Dependent Manner

**Figure 6:**
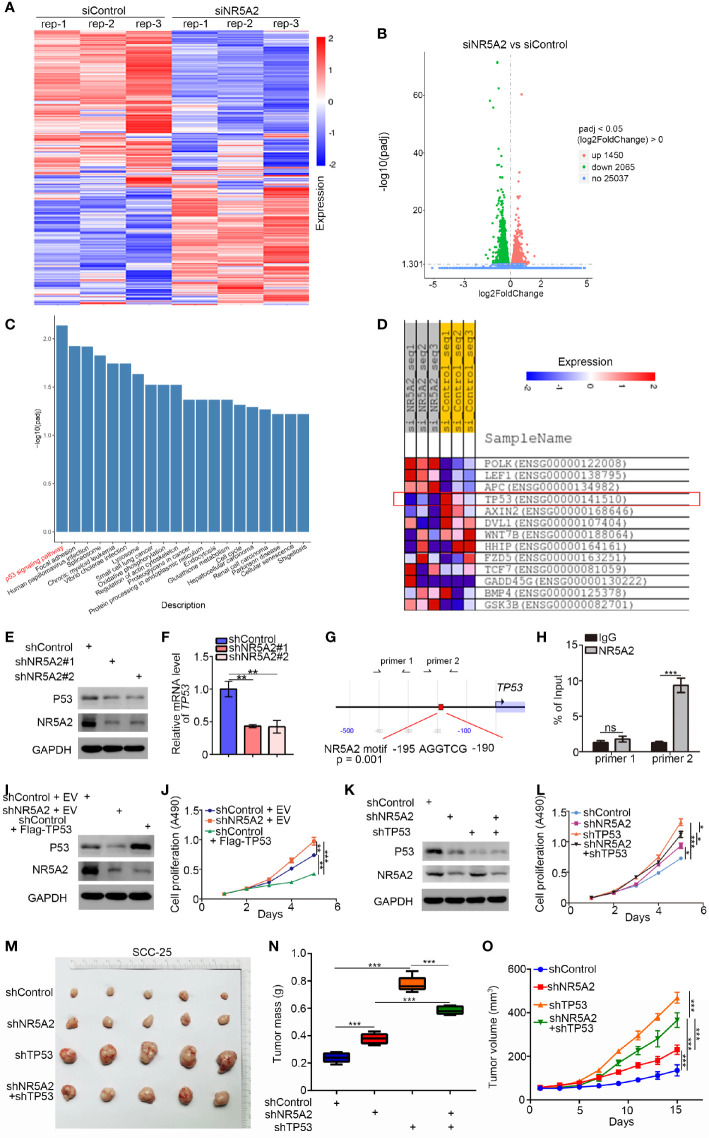
NR5A2 regulates HNSCC cell growth in a p53-dependent manner. **(A–D)** heatmap **(A)** and volcano plot **(B)** to show the differential expressed genes of FaDu cells infected by siControl or siNR5A2. **(C)** Enrichment analysis suggests that the differential genes between siControl and siNR5A2 groups may related to HNSCC. **(D)** RNA-Seq data suggest that knocking down NR5A2 correlation with the decrease of p53 expression. **(E, F)**, 48 h post-infection, FaDu cells were infected with shControl or shNR5A2 were harvested for Western blotting analysis **(E)** and RT-PCR analysis **(F)**. The data shown are the mean values ± SD from three replicates. **P < 0.01. **(G)**, the promoter region of TP53 was searched from The Eukaryotic Promoter Database exhibit that there was a consensus NR5A2 binding sites in the promoter region. **(H)**, the ChIP-qPCR analysis of FaDu cells. The data shown are the mean values ± SD from three replicates. ***P < 0.001. **(I, J)**, FaDu cells were infected with indicated shRNAs. After 48 h, cells were transfected with indicated plasmids (Empty vector (EV) and Flag-TP53) for another 24 h. Cells were harvested for Western Blot **(I)** and cell proliferation assay **(J)**. The data shown are the mean values ± SD from three replicates. **P < 0.01; ***P < 0.001. **(K–O)**, FaDu cells were infected with indicated shRNAs. 72 h post infection, cells were harvested for Western blotting **(K)**, MTS assay **(L)**, or xenografts assay **(M)**. The tumor volume was measured every three days **(N)**. Then the tumors were harvested, photographed **(M)** and weighed **(O)**. For MTS assay, data are shown as means ± SD (n = 3). *P < 0.05, ***P < 0.001. For xenograft assay, data are shown as means ± SD (n = 5). ***P < 0.001.

Given the opposing effects of NR5A2 on the growth of different HNSCC cell lines, we performed RNA sequencing analysis of FaDu cells to identify the mechanisms underlying our observations (**Figures 6A, B**). Gene ontology (GO) enrichment analysis demonstrated that the differentially expressed genes (DEGs) upon NR5A2 silencing were closely associated with head and neck carcinogenesis (**Figure 6C**). Interestingly, *TP53* was strongly downregulated in FaDu cells after NR5A2 silencing (**Figure 6D**). p53 downregulation in FaDu cells after NR5A2 silencing was confirmed (**Figures 6E, F**). *TP53* promoter analysis using the Eukaryotic Promoter Database (https://epd.epfl.ch//index.php) revealed the presence of NR5A2 binding sites (-195 AGG TCG -190) (**Figure 6G**). The Eukaryotic Promoter Database is an annotated non-redundant collection of eukaryotic POL II promoters, for which the transcription start site has been determined experimentally (10, 11). Subsequent ChIP-qPCR analyses confirmed the binding of NR5A2 to the *TP53* promoter (**Figure 6H**), suggesting that NR5A2 transcriptionally regulates *TP53* transcription in FaDu cells.

Notably, SCC-9 cells and SCC-15 cells exhibit extremely low p53 protein level, in contrast to FaDu and SCC-4 cells, which have high p53 level than non-malignant control cells reported previously (12). We found that down-regulation of NR5A2 was associated with significantly shorter DFS in the HNSCC patients with wild-type *TP53* but not in those in *TP53* mutation (**Supplementary Figures 2E, F**). P53 is the critical tumor suppressor to repress the cells proliferation in head and neck squamous carcinoma (13). Combined with the finding in the four different cancer cells with diverse p53 expression levels mentioned above, we hypothesized that NR5A2 regulated the cancer cells growth in a p53-dependent manner. Hence, we assessed the relevance of p53 in the ability of NR5A2 to regulate HNSCC cell growth. Forced the expression of p53 attenuated the ability of NR5A2 silencing to enhance cell proliferation in FaDu cells (**Figures 6I, J**). Silencing of both *NR5A2* and *TP53* decreased the proliferation of FaDu and SCC-4 cells to a greater extent than *TP53* silencing alone (**Figures 6K–O**, and **Supplementary Figures 3A, B**). Furthermore, our result also demonstrated that knockdown of NR5A2 decreased the expression of p53-related genes (e.g., *GADD45A, DDB2, SERPINE1, SHISA5, STEAP3, BAX, RRM2B, CD82*) associated with p53 pathway (14), which could be attenuated by *TP53* silencing in FaDu cells (**Supplementary Figure 3C**). These results suggest that the ability of NR5A2 to regulate HNSCC cell proliferation is p53-dependent.

### NR5A2 Regulates Glycolysis Independently of p53

**Figure 7:**
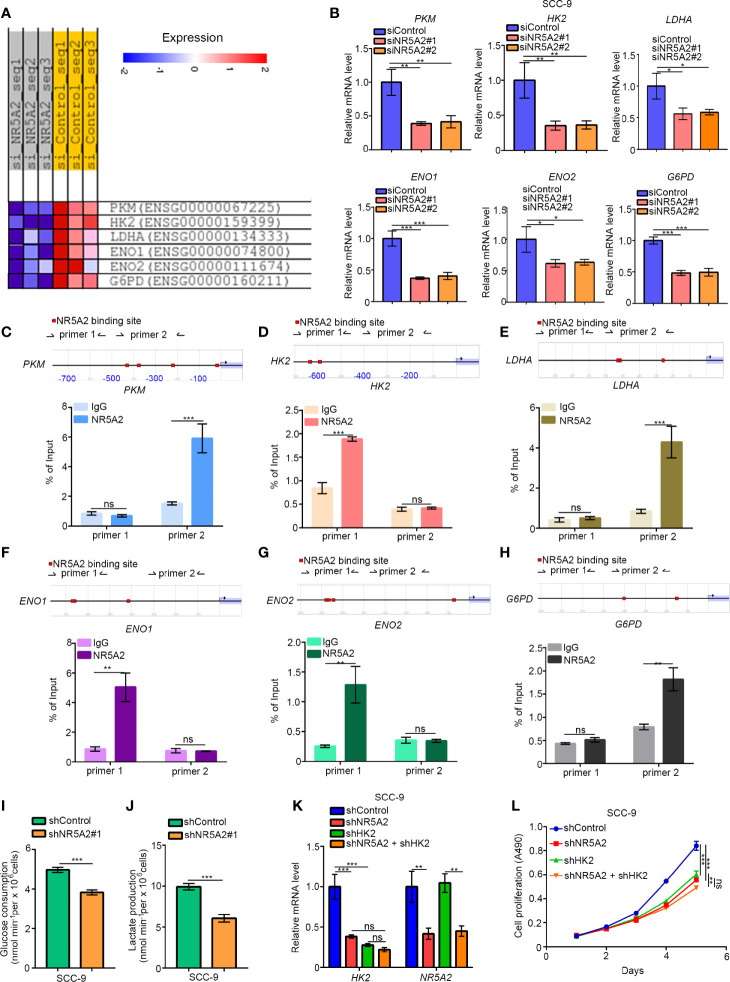
NR5A2 regulates glycolysis independently of p53. **(A)**, heatmap to show the genes related to glycolysis after knocking down of NR5A2 or not in FaDu cells. **(B)**, SCC-9 cells was infected with the specific siRNAs. After 48 h, cells were subjected to the RT-qPCR analysis. Data are shown as means ± SD (n = 3). *P < 0.05; **P < 0.01; ***P < 0.001. **(C–H)**, the ChIP-qPCR analysis of SCC-9 cells. Data are shown as means ± SD (n = 3). n.s, not significant; **P < 0.01; ***P < 0.001. **(I, J)**, SCC-9 cells were infected with indicated shRNAs. After 48 h, the spent medium of cells was changed with the fresh medium for another 24 h. The spent medium was collected for the measurement of glucose consumption **(I)** and lactate production **(J)**. The data shown are the mean values ± SD from three replicates. ***P < 0.001. **(K, L)**, SCC-9 cells were infected with indicated shRNAs. After 48 h, cells were harvested for RT-qPCR analysis and MTS assay. Data are shown as means ± SD (n = 3). n.s, not significant; **P < 0.01; ***P < 0.001.

Next, we explored the oncogenic mechanisms of NR5A2 in the p53 low expression cells. Although NR5A2 silencing in FaDu cells (p53 normal/high expression cells) significantly downregulated the expression of genes involved in glycolysis, such as *PKM*, *HK2*, *LDHA*, *ENO1*, *ENO2*, and *G6PD* (**Figure 7A**). The ability of NR5A2 silencing to downregulate these genes was also confirmed by RT-qPCR analysis in SCC-9 cells (p53 low expression cells) (**Figure 7B**). We also found that the promoters of these genes contained putative NR5A2 binding sites (the red highlight regions in the figure with P < 0.001) by analyzing the Eukaryotic Promoter Database (**Figures 7C–H**). Moreover, we have confirmed the NR5A2 binding to these promoter regions by ChIP-qPCR in SCC-9 cells (**Figures 7C–H**). Furthermore, NR5A2 silencing decreased glucose consumption and lactate production rates in SCC-9 cells (**Figures 7I, J**). It has been reported that dysregulated glycolysis is one of the mechanisms employed by cancer cells to facilitate growth (15). Thus, we were curious about whether NR5A2 promoted the cancer cells growth in p53 low expression cells through enhancing the glycolysis. Notably, HK2 is known to promote HNSCC cell growth (16). Here, we silenced HK2 and NR5A2 alone or in combination in SCC-9 cells (**Figure 7K**) and found that knockdown of HK2 or NR5A2 suppressed cancer cell growth (**Figure 7L**). Concurrent HK2 and NR5A2 silencing had stronger anti-growth effects than silencing of HK2 or NR5A2 alone (**Figure 7L**). In SCC-9 cells, NR5A2 silencing downregulated the expression of *MET* (**Supplementary Figure 3D**), which is reported to be the downstream target gene of HK2 and responsible for HK2 induced tumor initiation and progression (17, 18). This effect was diminished by concurrent knockdown of HK2 in SCC-9 cells (**Supplementary Figure 3D**). Therefore, these data suggest that NR5A2 regulates glycolysis in HNSCC cells independently of p53.

## Discussion

In this study, we revealed that the combined expression level of 12 TFs (XRCC5, NFYC, HOXA1, DLX5, RREB1, PIAS4, MAFB, NR5A2, LMO4, CRTC1, SRF and CNOT8) predicted RFS in HNSCC patients. We performed LASSO Cox analyses to eliminate the influence of confounding factors on the prognostic performance of the 12-TF signature. Importantly, a comparison of the prognostic value of the 12-TF signature and other prognostic markers indicated the superiority of the signature in predicting HNSCC patient survival. This is the first study to describe a TF signature as a prognostic factor in HNSCC. We also built a nomogram integrating the 12-TF signature score to predict 1, 3, 5-year RFS in HNSCC patients, which confirmed the high prognostic performance of the 12-TF signature in HNSCC. However, there are several limitations to this study. First, the nomogram was based on retrospective data from the TCGA database due to the incomplete clinical data of the validation dataset. Moreover, additional clinical factors should be integrated into the nomogram model to improve the accuracy and reliability of the model. Further, the prognostic value of the 12-TF signature needs to be confirmed in clinical samples.

Numerous studies have shown that these 12 TFs might be involved in malignant tumor development and progression. For example, XRCC5 has been shown to induce cyclooxygenase-2 expression as a complex with p300, thereby promoting colon cancer progression (19). Takenoyama et al. identified a point mutation in *NFYC* responsible for the induction of cytolytic T cell responses against human squamous cell lung carcinoma cells (20). It has been documented that DLX5 upregulation promoted ovarian cancer cells growth by activating IRS-2-AKT signaling (21). Additionally, MAFB sumoylation has been proposed as a novel therapeutic target for colorectal cancer (22). Schumacher et al. reported that aberrant CRTC1 activation promoted colon cancer growth by modulating PGE2 signaling (23). miRNA-99a has been shown to suppress liver cancer cell invasion and migration by suppressing HOXA1 expression (24). PIAS4 has been identified as a hypoxia signaling activator in pancreatic cancer cells (25). CNOT8 has been associated with progression-free survival in ovarian cancer patients (26). LMO4 has been shown to promote trastuzumab-resistance in HER2-positive breast tumors (27). RREB1-induced increasing of the lncRNA AGAP2-AS1 has been demonstrated to modulate the pancreatic cancer cells proliferation and migration partly *via* inhibiting ANGPTL4 and ANKRD1 (28). Arsenic trioxide has been shown to suppress liver cancer cells metastasis by targeting the SRF/MCM7 complex (29). Here, we focused on the role of NR5A2 in HNSCC due to its strong association with DFS and OS in HNSCC patients.

NR5A2 is a scaffolding protein recruiting DNA-binding molecules and thereby regulating the transcription of target genes. It belongs to the structure-class nuclear receptor family of TFs, which controls the expression of pancreatic enzymes involved in cholesterol and bile acid metabolism (30). However, the role of NR5A2 in cancer is controversial. NR5A2 has been shown to promote lung cancer development and progression by upregulating Nanog (31). Knockdown of NR5A2 in pancreatic cancer decreased the expression of FGB, TWIST, SNAIL, MMP9, MMP3, MMP2, and Vimentin, as well as increased the expression of the epithelial markers β-catenin and E-cadherin (32). In contrast, tumor-suppressive functions have also been ascribed to NR5A2. For instance, NR5A2 has been shown to suppress the development of KRAS (G12V)-driven mouse pancreatic intraepithelial neoplasms (33), and NR5A2 silencing suppressed the expression of molecules essential for cell proliferation, including cyclins D1/E1 and c-Myc (34). Furthermore, heterozygous mutations in NR5A2 rendered the mouse pancreas more susceptible to tissue damage, inhibited tissue regeneration, and cooperated with the mutant KRAS to promote tumor progression (35, 36). A recent meta-analysis has shown that NR5A2 functions as a tumor suppressor in pancreatic cancer (37). In this study, we demonstrated that NR5A2 downregulation was associated with poor prognosis in patients with HNSCC. Notably, we found that down-regulation of NR5A2 was associated with shorter disease-free survival time in the HNSCC patients with wild *TP53* subgroup but not in those harboring *TP53* mutations. Since P53 is the crucial tumor suppressor to repress the cells proliferation in head and neck squamous carcinoma (13). It was not surprising that NR5A2 suppressed the growth of HNSCC cell lines with normal/higher p53 expressed by upregulating *TP53* expression. On the contrary, due to glycolysis also important for the tumor growth, we pointed out that NR5A2 promoted cancer cell proliferation in HNSCC cell lines with *TP53* low expression by upregulating glycolytic enzymes.

**Figure 8:**
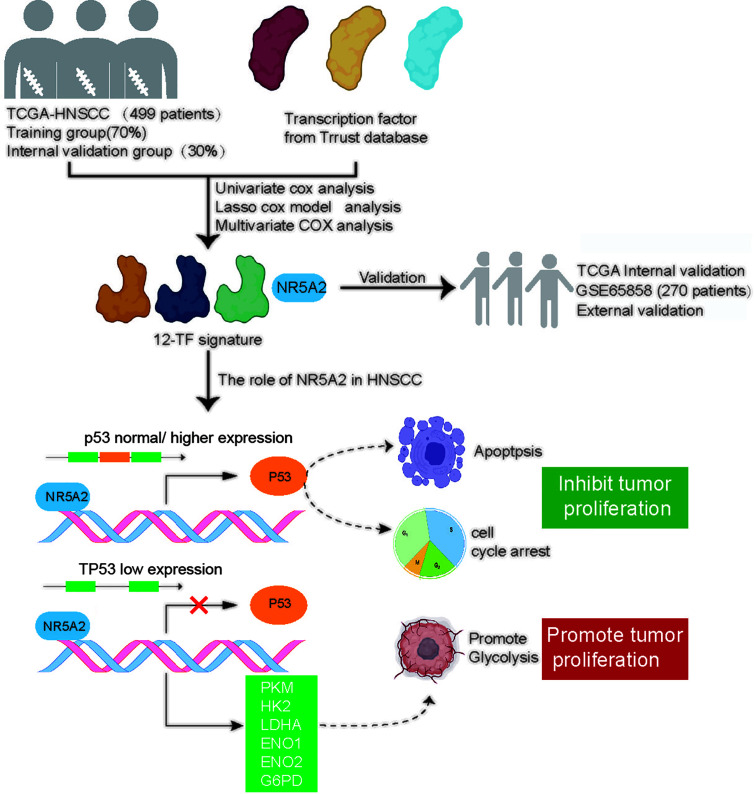
A hypothesis model depicted that a 12-TF signature was established to evaluate the prognosis of HNSCC. Additionally, we systemically studied the cancer biology effect of NR5A2, one of 12-TF signature, and revealed that NR5A2 blocked the tumor growth in HNSCC cell lines with p53 normal/higher expressed by increasing *TP53* expression, but promoted cancer cells proliferation in HNSCC cell lines with *TP53* loss/deletion *via* up-regulating the glycolysis-related enzymes.

In conclusion, we established a 12-TF signature that accurately and reliably predicts prognosis in patients with HNSCC. Additionally, we systemically investigated the role of NR5A2 in HNSCC and found that NR5A2 regulated HNSCC cell growth in a TP53 status-dependent manner. Therefore, NR5A2 may serve as a promising therapeutic target in HNSCC harboring loss-of-function *TP53* mutations (**Figure 8**).

## Data Availability Statement 

‘The original contributions presented in the study are included in the article/**Supplementary Material**. Further inquiries can be directed to the corresponding authors.

## Ethics Statement 

The animal study was reviewed and approved by Tongji Medical College, Huazhong University of Science and Technology.

## Author Contributions 

KZ: Methodology, Conceptualization. MX: Formal analysis, Methodology. XJ: Investigation, Methodology, Project administration, Writing - Original Draft. HJ: Funding acquisition, Project administration. All authors contributed to the article and approved the submitted version.

## Funding

This work was supported by grants from the Chinese National Natural Science Foundation Grant No. 81900947 (H.J.).

## Conflict of Interest

The authors declare that the research was conducted in the absence of any commercial or financial relationships that could be construed as a potential conflict of interest.

## Abbreviations

HNSCC, Head and neck squamous cell carcinoma; NR5A2, Nuclear receptor subfamily 5 group A member 2; TF, Transcription factors.
