# Supplementary material for: NR5A2 Is One of 12 Transcription Factors Predicting Prognosis in HNSCC and Regulates Cancer Cell Proliferation in a p53-Dependent Manner
Source: Front Oncol. 2021 Jul 1;11:691318. doi: 10.3389/fonc.2021.691318 (PMC8280457; doi:10.3389/fonc.2021.691318)
Supplement: Supplementary file 1 [file DataSheet_1.docx]

**NR5A2 is one of 12 transcription factors predicting prognosis in HNSCC and regulates cancer cell proliferation in a p53-dependent manner**

Kun Zhang, Ming Xiao, Xin Jin, Hongyan Jiang

**Supplementary methods**

**Nomogram construction**

Univariate and multivariate Cox proportional hazard analyses was performed using TFs risk score and other clinical factors. Hazard ratios (HR) and the corresponding 95% confidence interval (CI) was calculated by Cox proportional hazard models. Subsequently, we constructed a nomogram, combining the 12-TF signature and significant (P value < 0.05) clinicopathological risk factors identified in multivariate Cox regression analysis. The ability of the nomogram to predict clinical outcomes was evaluated using ROC, concordance index (C-index) calculation, and decision curve analysis (DCA).

**Gene set variation analysis (GSVA)**

We performed single -sample gene sets enrichment analysis (ssGSEA) on the basis of HNSCC mRNA dataset from TCGA via GSVA package to analyze the selected 12 TFs-relevant signaling pathways ^10^. All eligible patients were divided into a high-risk and low-risk groups according to cutoff value based on the average risk scores. *P-*values < 0.05 were considered statistically significant.

**The RT-qPCR assay，Chromatin immunoprecipitation (ChIP) and ChIP-qPCR**

Trizol reagent (#15596026, Invitrogen) extracted the total RNA from cells. PrimeScript™ RT kit (#RR047A, TAKARA, JPN) was used to reverse transcribed RNA sample (1μg). The TB Green™ Fast qPCR Mix kit (#RR430A, TAKARA, JPN) was used to perform RT-qPCR. All values were normalized by GAPDH, and the fold change was quantified using the 2-ΔCt method. The primer sequences of RT-qPCR and were shown in Table S1. ChIP was performed with the Chromatin Extraction Kit (#ab117152, Abcam) and ChIP Kit Magnetic - One Step (#ab156907, Abcam) according to the manufacturers standard protocol. The puriﬁed DNA was analyzed as same as RT-qPCR. Primers used for ChIP-qPCR were shown in Table S2.

**Colony formation assay and MTS assay**

For colony formation assay, 500 cells were added into 6-well plates and cultured for 2 weeks. After that, methanol was used to fix the colony for 0.5 h and 1% Crystal Violet was applied to stained the colony for 30 mins. The number of colonies was calculated. All assays were performed in triplicate. For MTS assay, (2000 cells were added into a 96-well plate and then 20 μl of MTS reagent (#ab197010, Abcam, USA) was added to each well for 60 mins. The absorbance of each well was measured at 490 nm with a microplate reader.

**Xenografts assay**

BALB/c-nu mice (4–5 weeks old) were obtained from Vitalriver (Beijing, China). The procedures were carried out following the guidelines formulated by the National Institutes of Health of China and approved by Ethical Committee on Animal Experiments at the Huazhong University of Science and Technology in Wuhan, China. Tumor cells infected with different shRNAs were subcutaneously injected into the left dorsal side of BALB/c-nu mice. Digital vernier calipers were used to evaluate tumor size every three days. The mice were sacrificed and the xenografts were excised and weighed.


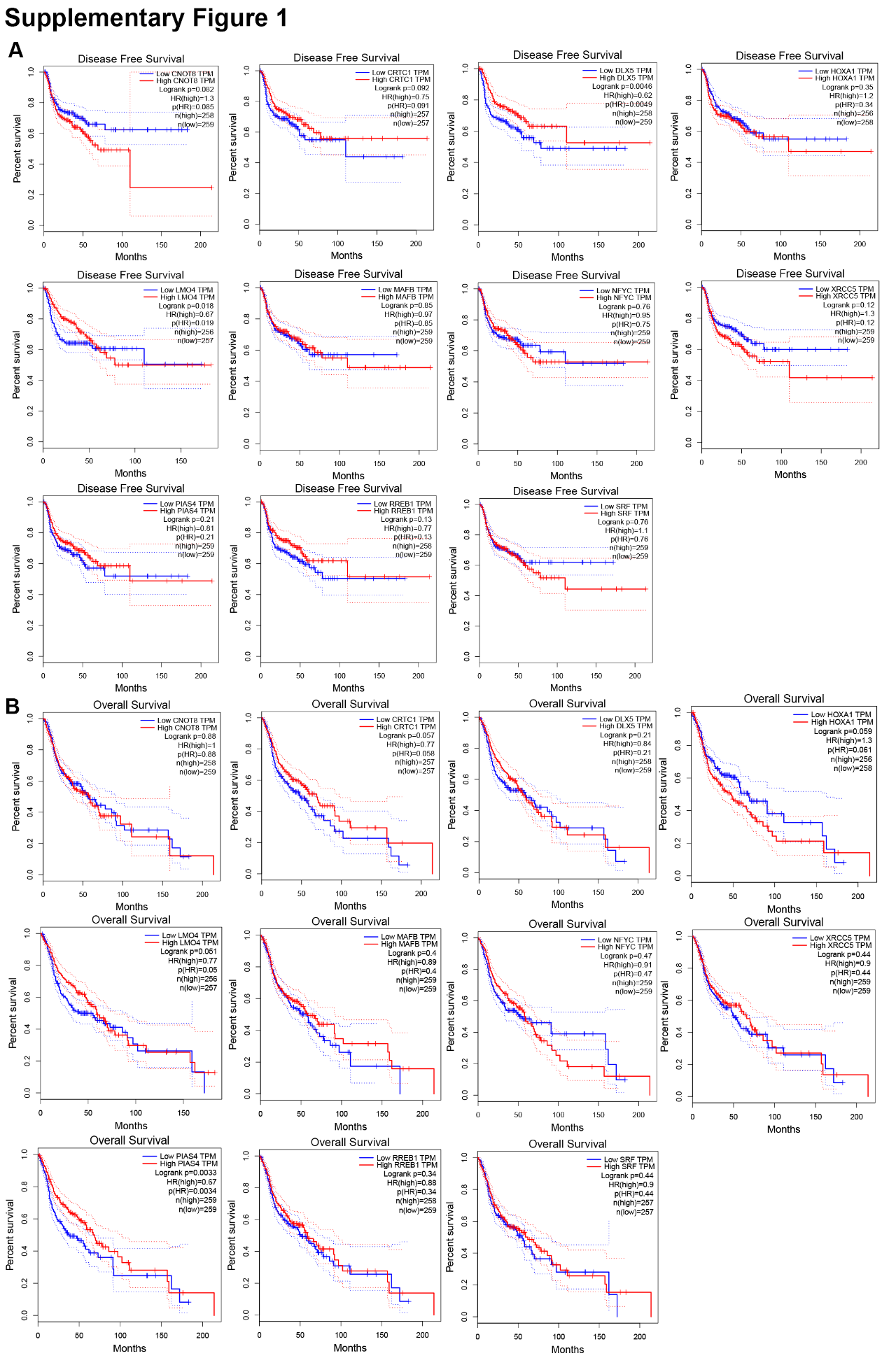


**Supplementary Figure 1**. **A**. The disease-free survival rate in high/low indicated genes were analyzed by the GEPIA web tool. **B**. The overall survival rate in high/low indicated genes were analyzed by the GEPIA web tool


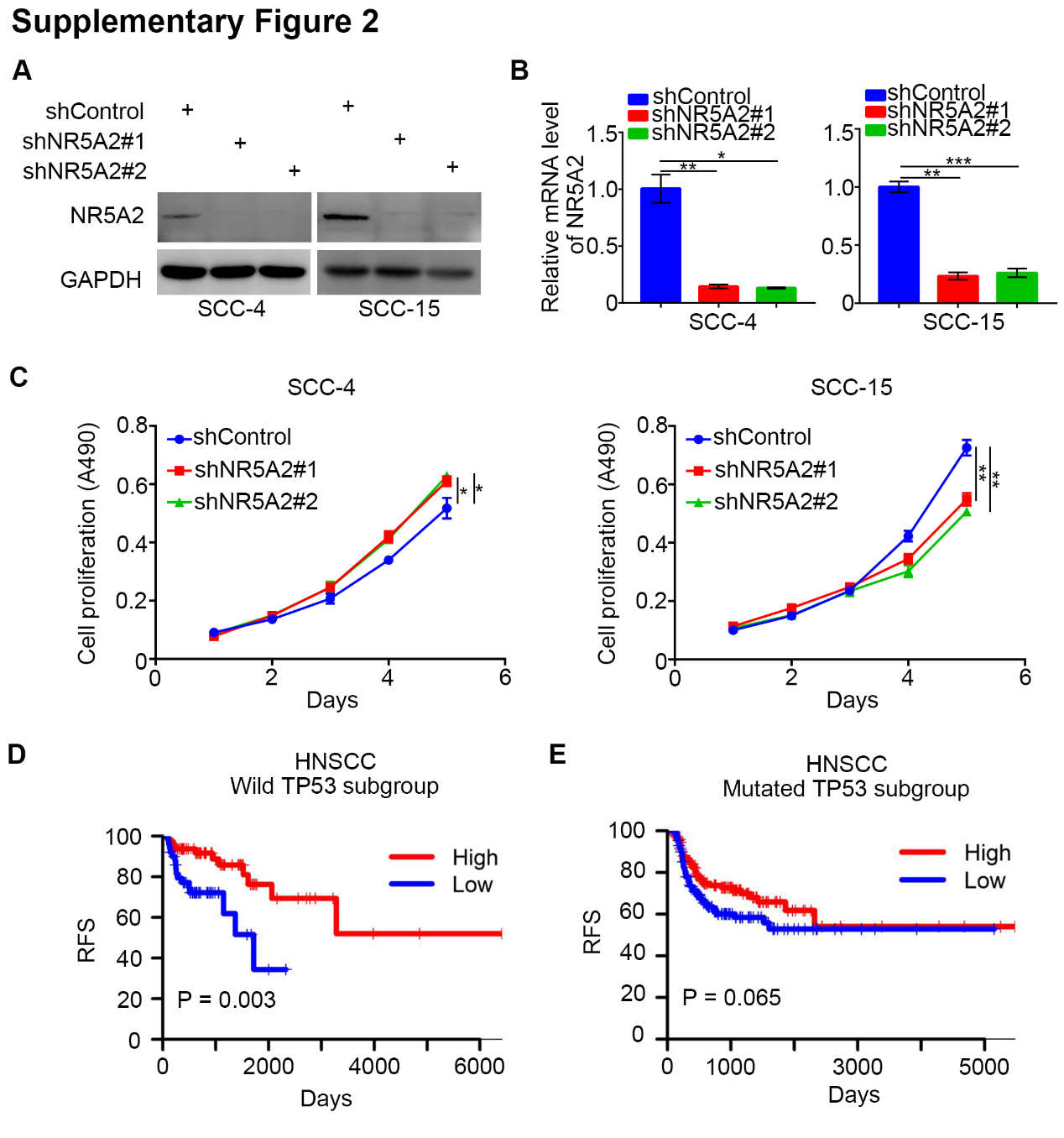


**Supplementary Figure 2. A-C,** SCC-4 and SCC-15 cells were transfected indicated shRNAs for 48 h. Cells were harvested for western blotting analysis (A), RT-qPCR analysis (B) and MTS assay (C). Data presented as the mean ± SD of three independent experiments. *, p < 0.05; **, p < 0.01. **D** and **E**, Kaplan-Meier analyses of wild-type TP53 subgroup (D) and mutated TP53 subgroup (E) of HNSCC patients in TCGA. In each analysis, the median of NR5A2 expression level was used as a cutoff value for classification of patients into high- and low- expression groups.


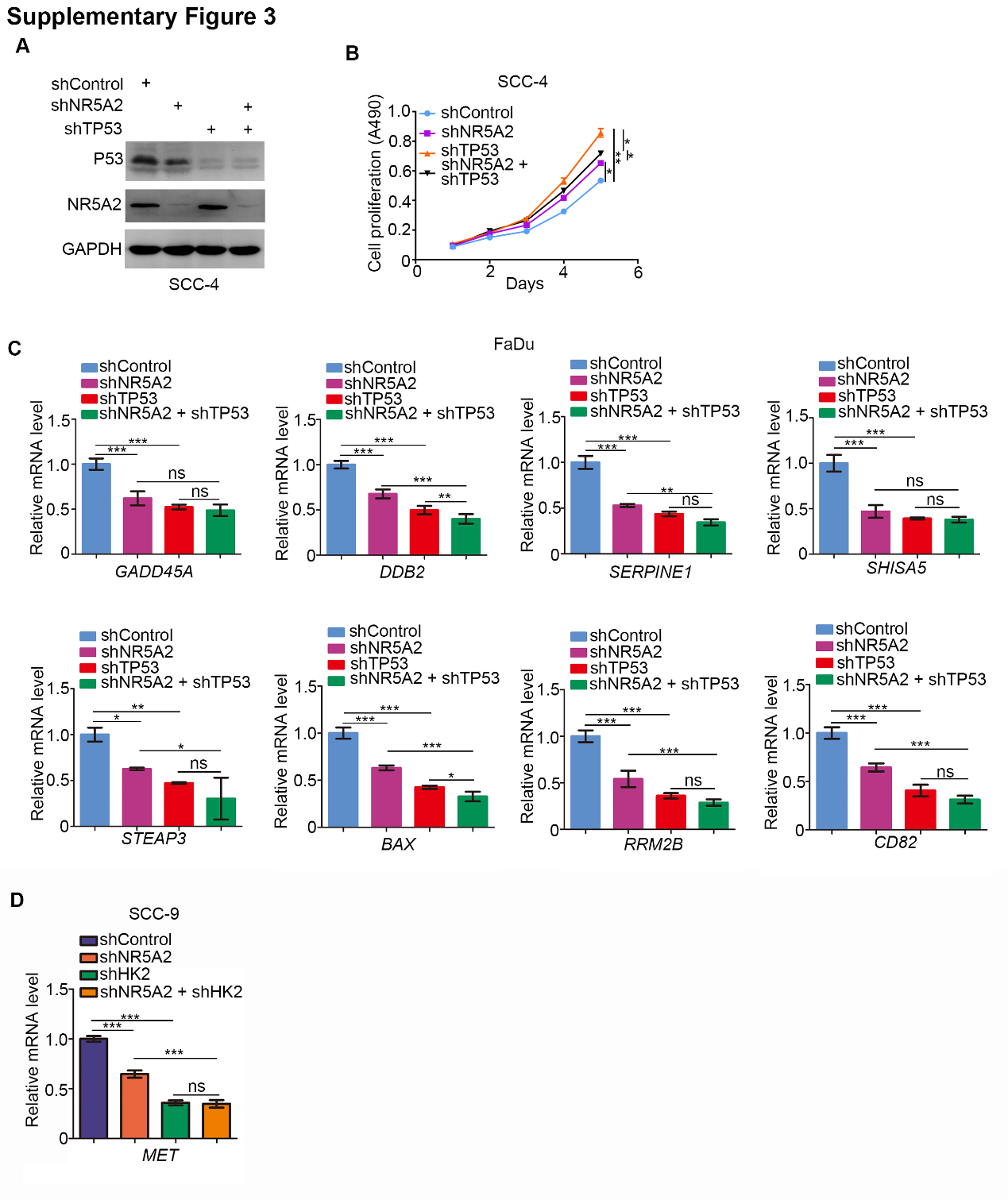


**Supplementary Figure 3. A** and **B,** SCC-4 cells were infected with indicated shRNAs. After 48 h, cells were harvested for Western Blot (A) and cell proliferation assay (B). The data shown are the mean values ± SD from three replicates. *, P < 0.05; **, P < 0.01. **C**, FaDu cells were infected with indicated shRNAs. After 48 h, cells were harvested for RT-qPCR analysis. The data shown are the mean values ± SD from three replicates. Ns, not significant; *, P < 0.05; **, P < 0.01; ***, P < 0.001. D, SCC-9 cells were infected with indicated shRNAs. After 48 h, cells were harvested for RT-qPCR analysis. The data shown are the mean values ± SD from three replicates. Ns, not significant; ***, P < 0.001.

**Table S1: Sequences of RT-qPCR primers**

| **Species** | **Gene** | **Forward (5’-3’)** | **Reverse (5’-3’)** |
| --- | --- | --- | --- |
| Human | *GAPDH* | CCAGAACATCATCCCTGCCT | CCTGCTTCACCACCTTCTTG |
| Human | *NR5A2* | AACATCCCCCATTAGCATGA | AGGCTCATCTGGCTCACACT |
| Human | *TP53* | GTTCCGAGAGCTGAATGAGG | TCTGAGTCAGGCCCTTCTGT |
| Human | *PKM* | AGTACCATGCGGAGACCATC | GCGTTATCCAGCGTGATTTT |
| Human | *HK2* | TCTATGCCATCCCTGAGGAC | TCTCTGCCTTCCACTCCACT |
| Human | *LDHA* | GGCCTGTGCCATCAGTATCT | CGCTTCCAATAACACGGTTT |
| Human | *ENO1* | TGATCGAGATGGATGGAACA | CGCCATTGATGACATTGAAC |
| Human | *ENO2* | GAAGAAAAGGCCTGCAACTG | CCAGGTCAGCAATGAATGTG |
| Human | *G6PD* | GAGGCCGTGTACACCAAGAT | AGCAGTGGGGTGAAAATACG |
| Human | *GADD45A* | ACGAGGACGACGACAGAGAT | GCAGGATCCTTCCATTGAGA |
| Human | *DDB2* | TCAAGGACAAACCCACCTTC | GTGACCACCATTCGGCTACT |
| Human | *SERPINE1* | CTCTCTCTGCCCTCACCAAC | GTGGAGAGGCTCTTGGTCTG |
| Human | *SHISA5* | TGTCCAGATTTCTGCTGTGG | GCAGGTGAAGCAGATGATGA |
| Human | *STEAP3* | CTTTCCAAGAGGAGGCAGTG | TTGGACTCACGATGCTGAAG |
| Human | *BAX* | TTTGCTTCAGGGTTTCATCC | CAGTTGAAGTTGCCGTCAGA |
| Human | *RRM2B* | TAAACAGGCACAGGCTTCCT | CGCTCCACCAAATTTTCATT |
| Human | *CD82* | GGGCTCAGCCTGTATCAAAG | ACATAGGCCCCCATCCTAAG |
| Human | *MET* | CAGGCAGTGCAGCATGTAGT | GATGATTCCCTCGGTCAGAA |

**Table S2: Sequences of ChIP-qPCR primers**

| **Species** | **Gene** | **Forward (5’-3’)** | **Reverse (5’-3’)** |
| --- | --- | --- | --- |
| Human | *TP53 primer 1* | AGGATCCAGCTGAGAGCAAA | GAGGGTGCAGAGTCAGGATT |
| Human | *TP53 primer 2* | TCCCCAACTCCATTTCCTTT | TGGACGGTGGCTCTAGACTT |
| Human | *PKM primer 1* | TGCAGGATTCCAGACCCTAC | CCTCGGAGACTGAGCTGAAC |
| Human | *PKM primer 2* | CCGAGAGCCAAGAAAAGACA | GCGGAAGGACACAGATTCAG |
| Human | *HK2 primer 1* | CCATGTCCGGCTGATTTATT | CCCCGTTTTACAGATGAGGA |
| Human | *HK2 primer 2* | ACAGTGTCTCTCCGCACAGG | CTGAGATGGGACGTGTGGT |
| Human | *LDHA primer 1* | GAGGCTGAGGCAGGAGATTC | CCCGAAGTGACTGGGACTAC |
| Human | *LDHA primer 2* | TTCACTGTGAGTGGGAGCTG | CTCAGGAAGGCTTGGATCTG |
| Human | *ENO1 primer 1* | CAGCAAGGAGAAAGGGACAG | GCGTCGAGTCGTTGAGAGG |
| Human | *ENO1 primer 2* | CCTATCTGGGGCCAGAGTTT | CGTCACTCATTCCCTCACCT |
| Human | *ENO2 primer 1* | ATTACAGGGGTGAGCCACAG | GGCCTCAGGTTGTGATGAAT |
| Human | *ENO2 primer 2* | GTTGGAGAGACTTGCGAAGG | CGACCCACTCGACAGTAACA |
| Human | *G6PD primer 1* | CTGTGGGATCCGGAAGTAAA | GGCGTGCTTATCATTACCG |
| Human | *G6PD primer 2* | AGGGACAGCCCAGAGGAG | CTGCTCTGCATCCCCAAT |

**Table S3: Sequences of gene-specific shRNAs and siRNAs.**

| shNR5A2-1 | 5′- CCGGGCGTTGTCCTTACTGTCGTTTCTCGAGAAACGACAGTAAGGACAACGCTTTTT-3′ |
| --- | --- |
| shNR5A2-2 | 5′- CCGGCCGAGTCCATAATGGGCTATTCTCGAGAATAGCCCATTATGGACTCGGTTTTT-3′ |
| siNR5A2-1 | CATGGCCTATTTGCAGCAA |
| siNR5A2-2 | CCTGGTTACTGGGCAACAA |

**Table S4. Clinical features of contained cases.**

| **Characteristics** | **TCGA-HNSCC (%)** | **GSE65858 (%)** |
| --- | --- | --- |
| **A18_Sex** |  |  |
| FEMALE | 133(26.65) | 47 (17.41) |
| MALE | 366(73.35) | 223 (82.59) |
| **age_at_initial_pathologic_diagnosis** |  |  |
| <50 | 76(15.23) | 41 (15.19) |
| >=50 | 423(84.77) | 229 (84.81) |
| **number_pack_years_smoked** |  |  |
| <30 | 86 (15.23) | 106 (39.26) |
| >=30 | 200 (40.08) | 162 (60.00) |
| **A5_M** |  |  |
| M0 | 479(95.99) | 263 (97.41) |
| M1 | 4(0.8) | 7 (2.59) |
| MX | 16(3.21) |  |
| **A3_T** |  |  |
| T1 | 31(6.21) | 35 (12.96) |
| T2 | 143(28.66) | 80 (29.63) |
| T3 | 139(27.86) | 58 (21.48) |
| T4 | 178(35.7) | 97 (35.93) |
| TX | 8(1.6) |  |
| **A4_N** |  |  |
| N0 | 254(50.9) | 94 (34.81) |
| N1 | 79(15.83) | 32 (11.85) |
| N2 | 142(28.5) | 132 (48.89) |
| N3 | 6(1.2) | 12 (4.44) |
| NX | 18(3.61) |  |
| **A6_Stage** |  |  |
| Stage I | 24(4.81) | 18 (6.67) |
| Stage II | 88(17.64) | 37 (13.70) |
| Stage III | 91(18.24) | 37 (13.70) |
| Stage IV | 296(57.3) | 178 (65.93) |
| **A7_Grade** |  |  |
| G1 | 65(13.03) |  |
| G2 | 302(60.52) |  |
| G3 | 110(22.04) |  |
| G4 | 1(0.2) |  |
| GX | 19(3.81) |  |
| Not Available1 | 2(0.4) |  |
| **lymphovascular_invasion_present** |  |  |
| NO | 218(43.69) |  |
| Not Available2 | 159(31.86) |  |
| YES | 122(24.45) |  |
| **margin_status** | |  |
| Close | 44(8.82) |  |
| Negative | 349(69.94) |  |
| Not Available3 | 48(9.62) |  |
| Positive | 58(11.62) |  |
| **perineural_invasion_present** |  |  |
| NO | 183(36.67) |  |
| Not Available4 | 148(29.66) |  |
| YES | 168(33.67) |  |
| **race_list.race** | |  |
| AMERICAN INDIAN OR ALASKA NATIVE | 2(0.4) |  |
| ASIAN | 11(2.2) |  |
| BLACK OR AFRICAN AMERICAN | 43(8.62) |  |
| Unknown | 14(2.8) |  |
| WHITE | 429(85.97) |  |
| **anatomic_neoplasm_subdivision** |  |  |
| Alveolar Ridge | 17(3.41) |  |
| Base of tongue | 20(4.01) |  |
| Buccal Mucosa | 21(4.21) |  |
| Floor of mouth | 59(11.82) |  |
| Hard Palate | 7(1.4) |  |
| Hypopharynx | 10(2) |  |
| Larynx | 111(22.24) |  |
| Lip | 3(0.6) |  |
| Oral Cavity | 80(16.03) |  |
| Oral Tongue | 128(25.65) |  |
| Oropharynx | 8(1.6) |  |
| Tonsil | 35(7.01) |  |
| **alcohol_history_documented** |  |  |
| NO | 159(31.86) |  |
| Not Available6 | 12(2.4) |  |
| YES | 328(65.73) |  |

**Table S5. Univariate Cox regression analysis and multivariate Cox regression analysis outcome in the light of TF risk score and a few clinic-based variables.**

| **Variable** | **HR_ unicox** | **HR.95L** | **HR.95H** | **pvalue** | **HR_ multicox** | **HR.95L** | **HR.95H** | **pvalue** |
| --- | --- | --- | --- | --- | --- | --- | --- | --- |
| Cancer_Status | 2.711083 | 2.258507 | 3.25435 | 9.90E-27 | 2.774726 | 2.269112 | 3.393002 | 2.69E-23 |
| Score | 2.718282 | 2.196936 | 3.363346 | 3.43E-20 | 2.217533 | 1.777151 | 2.767041 | 1.78E-12 |
| number_pack_years_smoked | 0.931015 | 0.882454 | 0.982248 | 0.008915 | 0.922867 | 0.873193 | 0.975367 | 0.004462 |
| alcohol_history_documented | 1.33117 | 1.091334 | 1.623712 | 0.004769 | 1.181774 | 0.961393 | 1.452674 | 0.112723 |
| radiation_therapy | 1.373827 | 1.013583 | 1.862107 | 0.040666 | 1.255916 | 0.916864 | 1.720347 | 0.155804 |
| Stage | 1.471997 | 1.192854 | 1.816463 | 0.000314 | 1.195807 | 0.901596 | 1.586024 | 0.214589 |
| T | 1.147798 | 1.012965 | 1.300578 | 0.030618 | 0.912776 | 0.778531 | 1.070171 | 0.260836 |
| N | 1.167762 | 1.035184 | 1.31732 | 0.011657 | 1.103541 | 0.959019 | 1.269841 | 0.168917 |
| margin_status | 1.34987 | 1.091053 | 1.670082 | 0.005739 | 0.959096 | 0.768579 | 1.196839 | 0.71165 |

**Table S6. Hazard ratios and 95% CIs and P values of 795 TFs across univariate Cox regression analysis.**

| **Characteristics** | **HR** | **CI 95%** | **P.value** |
| --- | --- | --- | --- |
| NR1D1 | 0.9999 | 0.99963-1.00018 | 0.48715 |
| HOXA10 | 1.00074 | 0.99917-1.00232 | 0.35424 |
| DDIT3 | 1.00016 | 0.99993-1.00039 | 0.18261 |
| BTG2 | 1 | 0.99993-1.00007 | 0.89748 |
| ZNF300 | 1.00009 | 0.99817-1.00201 | 0.93023 |
| TIAL1 | 1.00084 | 1.00044-1.00124 | 3.00E-05 |
| FUBP3 | 0.99984 | 0.9994-1.00028 | 0.47366 |
| FUBP1 | 1.00009 | 0.99976-1.00042 | 0.58926 |
| BRIP1 | 1.00016 | 0.99919-1.00112 | 0.75155 |
| ENO1 | 1.00001 | 1.00001-1.00002 | 0.00063 |
| EOMES | 1.00013 | 0.99761-1.00265 | 0.921 |
| ZBTB7A | 0.99971 | 0.99936-1.00005 | 0.09779 |
| ILF3 | 1.00002 | 0.99992-1.00012 | 0.69487 |
| ILF2 | 1.00012 | 1.00004-1.00021 | 0.00569 |
| SETBP1 | 0.99983 | 0.99921-1.00046 | 0.59732 |
| POU3F1 | 0.99993 | 0.99947-1.00038 | 0.75266 |
| DMAP1 | 1.00017 | 0.99983-1.00052 | 0.32264 |
| XRCC5 | 1.0001 | 1-1.0002 | 0.04104 |
| XRCC6 | 1.00005 | 1.00002-1.00009 | 0.00182 |
| MEIS2 | 0.99896 | 0.99774-1.00018 | 0.09389 |
| MEIS1 | 0.99917 | 0.99843-0.99992 | 0.03084 |
| HES1 | 0.99986 | 0.99963-1.0001 | 0.24846 |
| BCL6 | 0.99961 | 0.99935-0.99987 | 0.00313 |
| ASXL1 | 1.00006 | 0.99974-1.00038 | 0.70927 |
| HIRA | 1.00064 | 0.99962-1.00165 | 0.21952 |
| CRABP2 | 0.99999 | 0.99995-1.00002 | 0.41245 |
| ZNF410 | 1.00555 | 0.99998-1.01115 | 0.05099 |
| TFAP4 | 0.99935 | 0.99845-1.00026 | 0.16173 |
| EAF1 | 1.00015 | 0.99952-1.00078 | 0.64435 |
| FOXF1 | 0.99725 | 0.99445-1.00005 | 0.05458 |
| FOXF2 | 1.00154 | 1.0003-1.00279 | 0.01508 |
| GABPB2 | 1.00041 | 0.99934-1.00148 | 0.45017 |
| NOTCH3 | 0.99995 | 0.99991-0.99999 | 0.0081 |
| ZNF383 | 0.99977 | 0.99753-1.00201 | 0.83921 |
| EHMT2 | 1.00017 | 0.9999-1.00045 | 0.22003 |
| HTATIP2 | 1.00023 | 1.00003-1.00042 | 0.0207 |
| NFYC | 0.99926 | 0.99864-0.99988 | 0.02022 |
| NFYB | 1.00038 | 0.99943-1.00134 | 0.43254 |
| NFYA | 1.00065 | 0.99997-1.00133 | 0.06171 |
| SKIL | 0.99998 | 0.99982-1.00015 | 0.82885 |
| THRA | 0.9996 | 0.99911-1.00009 | 0.11058 |
| CREB5 | 1.00039 | 0.9998-1.00098 | 0.196 |
| CREB1 | 1.00045 | 0.99984-1.00106 | 0.14954 |
| AES | 0.99999 | 0.99994-1.00005 | 0.79522 |
| LHX2 | 0.99986 | 0.99839-1.00134 | 0.85556 |
| LHX4 | 1.00341 | 0.98618-1.02094 | 0.69994 |
| YBX1 | 1.00002 | 0.99998-1.00005 | 0.3732 |
| NCOA2 | 0.99975 | 0.99939-1.0001 | 0.16204 |
| NCOA3 | 1.00007 | 0.99989-1.00026 | 0.45411 |
| NCOA1 | 0.99921 | 0.9988-0.99962 | 0.00014 |
| NCOA6 | 0.99991 | 0.99968-1.00014 | 0.43107 |
| ERF | 1.00009 | 0.99979-1.00039 | 0.54549 |
| ERG | 0.99932 | 0.99831-1.00033 | 0.18658 |
| HAND2 | 0.99727 | 0.99166-1.00292 | 0.34325 |
| HOXA5 | 0.99905 | 0.99529-1.00284 | 0.62362 |
| HOXA4 | 0.99715 | 0.99045-1.00389 | 0.40609 |
| HOXA1 | 1.0048 | 1.00274-1.00687 | 0 |
| WWP1 | 1.00005 | 0.99969-1.00041 | 0.78669 |
| JARID2 | 1.00001 | 0.99975-1.00027 | 0.93159 |
| NFKBIB | 1.00027 | 0.99981-1.00072 | 0.24683 |
| NFKBIA | 1.00002 | 0.99996-1.00008 | 0.51661 |
| NFKBIZ | 0.99996 | 0.99977-1.00014 | 0.64965 |
| NR5A1 | 0.9997 | 0.99917-1.00022 | 0.25685 |
| DLX5 | 0.99917 | 0.99841-0.99992 | 0.031 |
| DLX3 | 0.99984 | 0.99935-1.00033 | 0.50921 |
| TP63 | 0.99999 | 0.99997-1.00002 | 0.70525 |
| WWTR1 | 0.99998 | 0.9998-1.00016 | 0.85266 |
| CNBP | 1.00004 | 0.99998-1.0001 | 0.22623 |
| PIR | 1.00012 | 0.99981-1.00043 | 0.44803 |
| MLXIPL | 0.99992 | 0.99839-1.00145 | 0.91763 |
| HNRNPR | 1.00015 | 0.9999-1.00039 | 0.24215 |
| MAML1 | 0.99975 | 0.99928-1.00022 | 0.30116 |
| TEAD1 | 0.99995 | 0.99975-1.00015 | 0.59641 |
| TEAD4 | 1.00023 | 0.99987-1.0006 | 0.21062 |
| FOXM1 | 1.00004 | 0.99988-1.0002 | 0.61316 |
| UBTF | 0.9999 | 0.99963-1.00018 | 0.49454 |
| RELB | 0.99991 | 0.99968-1.00014 | 0.43138 |
| RELA | 1.00005 | 0.99984-1.00026 | 0.61724 |
| HDGF | 1.00003 | 0.99997-1.00009 | 0.33834 |
| COPS5 | 1.00044 | 1.00021-1.00067 | 0.00014 |
| SSB | 1.00031 | 1.00011-1.00051 | 0.00202 |
| ZNF335 | 0.99993 | 0.99899-1.00086 | 0.87894 |
| PRDM2 | 0.99929 | 0.99881-0.99977 | 0.00377 |
| EGR2 | 1.00013 | 0.99979-1.00047 | 0.44116 |
| EGR3 | 1.00005 | 0.9997-1.00041 | 0.77474 |
| EGR1 | 1 | 0.99997-1.00002 | 0.86821 |
| ZEB1 | 0.99974 | 0.99927-1.00021 | 0.28166 |
| AATF | 1.00021 | 1.00003-1.00039 | 0.02467 |
| NFKB1 | 0.9998 | 0.99953-1.00007 | 0.14119 |
| NFKB2 | 1.00001 | 0.99985-1.00017 | 0.92283 |
| HOPX | 0.99997 | 0.99992-1.00002 | 0.23288 |
| NFATC1 | 0.99951 | 0.99877-1.00026 | 0.20005 |
| NRIP1 | 1.00004 | 0.99991-1.00017 | 0.54568 |
| FOS | 1 | 0.99998-1.00001 | 0.63972 |
| SEC14L2 | 1.00027 | 1.00009-1.00044 | 0.00288 |
| UPF1 | 0.99981 | 0.99961-1.00002 | 0.07717 |
| UPF2 | 1.00001 | 0.99945-1.00057 | 0.96967 |
| ISL1 | 0.99646 | 0.99223-1.0007 | 0.1012 |
| ZNF143 | 1.00146 | 0.99965-1.00327 | 0.11293 |
| ZNF148 | 0.99974 | 0.99933-1.00016 | 0.22031 |
| LYL1 | 0.99974 | 0.99678-1.00271 | 0.86274 |
| STAT5B | 1.0001 | 0.99974-1.00047 | 0.57673 |
| CTCF | 0.99976 | 0.99918-1.00034 | 0.41355 |
| ZNF423 | 0.99871 | 0.99728-1.00014 | 0.07775 |
| NUPR1 | 0.99995 | 0.99985-1.00006 | 0.38182 |
| FOXE1 | 0.99983 | 0.99969-0.99998 | 0.02348 |
| SKI | 0.99973 | 0.99948-0.99998 | 0.03632 |
| PHF8 | 0.99949 | 0.99898-0.99999 | 0.04666 |
| ATRX | 0.99969 | 0.99935-1.00003 | 0.07533 |
| PREB | 1.00024 | 0.99986-1.00063 | 0.21773 |
| VHL | 0.99991 | 0.99923-1.00059 | 0.79676 |
| CEBPZ | 1.00016 | 0.99968-1.00064 | 0.50759 |
| APBB1 | 0.99959 | 0.99881-1.00036 | 0.29859 |
| WDR5 | 1.00016 | 0.9999-1.00043 | 0.23119 |
| POU2AF1 | 0.99951 | 0.99905-0.99996 | 0.03437 |
| ZFHX3 | 0.99955 | 0.99896-1.00014 | 0.13552 |
| CBX8 | 1.00041 | 0.99899-1.00184 | 0.56969 |
| CBX7 | 0.99961 | 0.99912-1.00009 | 0.11422 |
| ERCC2 | 1.0005 | 0.99998-1.00102 | 0.06072 |
| CREG1 | 1.00003 | 0.99999-1.00006 | 0.1781 |
| ABL1 | 0.99984 | 0.99962-1.00007 | 0.16948 |
| HNRNPD | 1.00006 | 0.99994-1.00017 | 0.3502 |
| CTBP1 | 0.9999 | 0.99967-1.00013 | 0.37829 |
| EWSR1 | 1.00015 | 1.00004-1.00027 | 0.00969 |
| DNMT1 | 0.99995 | 0.99981-1.00009 | 0.46069 |
| HOXD9 | 1.00041 | 0.99908-1.00175 | 0.54414 |
| HOXD3 | 1.00033 | 0.95357-1.04938 | 0.98923 |
| KLF7 | 1.00014 | 0.99994-1.00034 | 0.16853 |
| KLF6 | 1.00004 | 0.99999-1.0001 | 0.13264 |
| KLF5 | 0.99999 | 0.99994-1.00003 | 0.53411 |
| KLF4 | 0.99997 | 0.9999-1.00004 | 0.37429 |
| KLF3 | 0.99977 | 0.99959-0.99995 | 0.01341 |
| KLF2 | 0.99949 | 0.99868-1.00031 | 0.22123 |
| KLF1 | 1.03375 | 0.98451-1.08545 | 0.18252 |
| KLF9 | 0.99982 | 0.99955-1.00009 | 0.18432 |
| KLF8 | 0.99986 | 0.99903-1.0007 | 0.74762 |
| REST | 0.99973 | 0.99912-1.00034 | 0.38208 |
| RARA | 1.00002 | 0.99945-1.00059 | 0.93825 |
| RARB | 0.99874 | 0.99752-0.99996 | 0.04378 |
| RARG | 0.99982 | 0.99968-0.99997 | 0.01542 |
| SALL4 | 0.99944 | 0.99405-1.00487 | 0.84032 |
| HBP1 | 0.99974 | 0.99936-1.00012 | 0.1796 |
| MXI1 | 0.9999 | 0.99949-1.00032 | 0.6506 |
| MEN1 | 1.00019 | 0.99982-1.00057 | 0.31884 |
| AHR | 1.00004 | 0.99998-1.0001 | 0.23493 |
| UHRF1 | 1.00027 | 0.99988-1.00065 | 0.17149 |
| RB1 | 1.00003 | 0.99982-1.00025 | 0.7569 |
| FHL2 | 1.00014 | 1.00004-1.00024 | 0.00631 |
| PAWR | 1.00023 | 0.99995-1.00052 | 0.10881 |
| TP53BP1 | 0.99991 | 0.99932-1.0005 | 0.7578 |
| DDB2 | 1.00024 | 0.9999-1.00059 | 0.16911 |
| EHF | 0.99987 | 0.99978-0.99996 | 0.00407 |
| ARNTL | 1.00042 | 0.99952-1.00131 | 0.36302 |
| MYBBP1A | 1.0001 | 0.99989-1.00032 | 0.34337 |
| HDAC1 | 1.00003 | 0.99989-1.00016 | 0.69024 |
| HDAC3 | 1.00063 | 1.00025-1.00101 | 0.00108 |
| HDAC2 | 1.00014 | 0.99991-1.00038 | 0.23906 |
| HDAC5 | 0.99991 | 0.99961-1.0002 | 0.53104 |
| HDAC4 | 0.99915 | 0.99851-0.99978 | 0.00861 |
| HDAC7 | 0.99993 | 0.99961-1.00024 | 0.64957 |
| HDAC9 | 1.00049 | 0.99995-1.00103 | 0.07491 |
| SRCAP | 0.99984 | 0.99968-1.00001 | 0.06932 |
| CEBPB | 1.00007 | 0.99994-1.0002 | 0.31745 |
| CEBPA | 0.99941 | 0.99887-0.99996 | 0.03443 |
| CEBPG | 1.00008 | 0.99994-1.00023 | 0.25091 |
| CEBPE | 0.99927 | 0.99248-1.00611 | 0.83462 |
| CEBPD | 0.9999 | 0.99974-1.00005 | 0.20048 |
| HDAC11 | 1.00018 | 0.99954-1.00082 | 0.58715 |
| HIF3A | 0.99641 | 0.99233-1.00051 | 0.08579 |
| XPC | 0.99952 | 0.99874-1.0003 | 0.22396 |
| DENND4A | 0.99948 | 0.99863-1.00034 | 0.23901 |
| TRERF1 | 0.99979 | 0.99927-1.00031 | 0.42535 |
| SMAD4 | 0.99975 | 0.9993-1.0002 | 0.2701 |
| SMAD7 | 1.00016 | 0.9996-1.00071 | 0.58026 |
| SMAD1 | 0.99934 | 0.9986-1.00007 | 0.07817 |
| SMAD2 | 0.99992 | 0.99967-1.00017 | 0.53787 |
| SMAD3 | 0.99998 | 0.99986-1.00011 | 0.80902 |
| RFX5 | 0.99987 | 0.99946-1.00027 | 0.51553 |
| RFX1 | 0.99871 | 0.99715-1.00027 | 0.10491 |
| RFX2 | 1.00051 | 0.99958-1.00144 | 0.2856 |
| RFX3 | 0.9987 | 0.9966-1.00081 | 0.22616 |
| CDC5L | 1.00045 | 1.00002-1.00089 | 0.04253 |
| BRD7 | 1.00021 | 0.99979-1.00064 | 0.33008 |
| SLA2 | 0.99974 | 0.99779-1.0017 | 0.79646 |
| GTF2F1 | 1.00019 | 0.99991-1.00046 | 0.17939 |
| CUX1 | 0.99988 | 0.99976-1 | 0.0456 |
| TOB1 | 0.99975 | 0.99946-1.00004 | 0.09006 |
| CTNNBIP1 | 0.99997 | 0.99984-1.0001 | 0.67735 |
| CITED2 | 1.00008 | 0.99993-1.00024 | 0.29848 |
| EBF3 | 0.99912 | 0.99731-1.00094 | 0.34503 |
| EBF1 | 0.99908 | 0.99765-1.00051 | 0.20863 |
| FOXH1 | 1.0001 | 0.99612-1.00408 | 0.96256 |
| HLTF | 1.00005 | 0.9998-1.0003 | 0.6842 |
| RUNX1T1 | 0.99856 | 0.99565-1.00148 | 0.33441 |
| TFAP2A | 1.00016 | 0.99999-1.00032 | 0.06576 |
| TFAP2C | 0.99991 | 0.99958-1.00023 | 0.57354 |
| RREB1 | 0.99962 | 0.99938-0.99985 | 0.00143 |
| CDCA7L | 1.00035 | 0.99977-1.00093 | 0.24062 |
| ZHX2 | 0.99973 | 0.9993-1.00017 | 0.23507 |
| DEK | 1.00009 | 0.99999-1.00018 | 0.08638 |
| TBPL1 | 1.00121 | 1.00046-1.00196 | 0.00154 |
| FUS | 1.00003 | 0.99994-1.00013 | 0.47391 |
| ONECUT2 | 1.00083 | 0.99832-1.00335 | 0.51494 |
| HSF2 | 1.00013 | 0.99881-1.00146 | 0.8431 |
| HSF4 | 1.00117 | 0.99996-1.00239 | 0.05777 |
| RORC | 0.99942 | 0.99861-1.00023 | 0.16166 |
| RORA | 0.99953 | 0.99905-1 | 0.05122 |
| TRRAP | 0.99977 | 0.99954-1 | 0.05512 |
| ZMYND11 | 0.99985 | 0.99947-1.00024 | 0.46266 |
| TAF5 | 1.00246 | 0.99917-1.00578 | 0.14333 |
| TAF1 | 0.99962 | 0.99909-1.00014 | 0.15553 |
| FOSB | 1.00001 | 0.99997-1.00004 | 0.68015 |
| PDCD11 | 1 | 0.99973-1.00028 | 0.97355 |
| CREM | 1.00023 | 0.99924-1.00123 | 0.64493 |
| TRIM28 | 1 | 0.99994-1.00006 | 0.8947 |
| TRIM22 | 1.00003 | 0.99994-1.00012 | 0.5299 |
| GTF3A | 1.00018 | 1.00001-1.00034 | 0.03446 |
| IFI16 | 1.00003 | 0.99997-1.0001 | 0.30511 |
| LRRFIP1 | 1 | 0.99987-1.00013 | 0.9736 |
| POU3F2 | 1.00329 | 0.99305-1.01365 | 0.52995 |
| SP100 | 1.00021 | 1.00003-1.0004 | 0.02241 |
| ZNF217 | 0.99964 | 0.99931-0.99996 | 0.02903 |
| NR2E3 | 0.9845 | 0.91529-1.05895 | 0.67449 |
| SMG6 | 0.99907 | 0.99835-0.9998 | 0.01253 |
| SF1 | 0.99989 | 0.99971-1.00007 | 0.23743 |
| SCD5 | 0.99988 | 0.99955-1.00021 | 0.4776 |
| ETS2 | 0.99999 | 0.99993-1.00004 | 0.62963 |
| ETS1 | 1.00004 | 0.99994-1.00015 | 0.41848 |
| CBFA2T3 | 0.99871 | 0.99724-1.00018 | 0.08549 |
| JUN | 0.99999 | 0.99995-1.00003 | 0.68452 |
| NPAS3 | 0.99029 | 0.97797-1.00278 | 0.12702 |
| NPAS2 | 0.99977 | 0.99924-1.0003 | 0.39428 |
| PURA | 0.99885 | 0.99792-0.99978 | 0.01502 |
| NFAT5 | 0.99973 | 0.99935-1.00012 | 0.17763 |
| MXD1 | 0.99996 | 0.99987-1.00004 | 0.30847 |
| MED23 | 0.9997 | 0.9987-1.00071 | 0.56361 |
| NONO | 1.00006 | 0.99998-1.00014 | 0.16689 |
| PARP1 | 0.99998 | 0.99986-1.0001 | 0.74631 |
| TFDP1 | 1.00001 | 0.99995-1.00006 | 0.83062 |
| ANKRD1 | 1.00002 | 0.99991-1.00013 | 0.70576 |
| BIN1 | 1.00001 | 0.99996-1.00006 | 0.68745 |
| FOXP2 | 0.9988 | 0.99775-0.99984 | 0.02418 |
| FOXP3 | 0.99989 | 0.99886-1.00092 | 0.83567 |
| FOXP1 | 0.99995 | 0.99965-1.00026 | 0.76702 |
| MYCN | 0.99686 | 0.99354-1.00019 | 0.06458 |
| ECD | 1.00153 | 1.00052-1.00254 | 0.00287 |
| ARID3A | 1.00088 | 0.99997-1.0018 | 0.05703 |
| BTF3 | 1.00002 | 0.99997-1.00007 | 0.39207 |
| FOXO3 | 0.99962 | 0.99928-0.99995 | 0.02377 |
| FOXO1 | 0.99984 | 0.99938-1.00029 | 0.48172 |
| FOXO4 | 0.99935 | 0.99845-1.00025 | 0.15896 |
| PIAS2 | 0.99988 | 0.99905-1.0007 | 0.77191 |
| PIAS3 | 0.99997 | 0.99961-1.00033 | 0.88298 |
| PIAS1 | 0.99969 | 0.99881-1.00058 | 0.49974 |
| PIAS4 | 1.00087 | 1.00002-1.00172 | 0.04582 |
| HMGB2 | 1.00006 | 0.99996-1.00016 | 0.25064 |
| BARD1 | 1.00012 | 0.99902-1.00122 | 0.83437 |
| TTF2 | 0.99995 | 0.99956-1.00033 | 0.78948 |
| POU5F1 | 1.00252 | 0.99985-1.0052 | 0.06457 |
| HOXD13 | 0.99956 | 0.99826-1.00085 | 0.50222 |
| ZNF160 | 0.99983 | 0.99881-1.00085 | 0.74085 |
| GTF2A1 | 0.9999 | 0.99943-1.00037 | 0.68001 |
| BPTF | 0.9996 | 0.99924-0.99995 | 0.02689 |
| TCF12 | 0.99992 | 0.9996-1.00023 | 0.60514 |
| TCF19 | 1.00026 | 1.00002-1.0005 | 0.0325 |
| CREB3 | 1.00003 | 0.99992-1.00014 | 0.60212 |
| ZFP36 | 0.99997 | 0.99994-1.00001 | 0.12592 |
| TFCP2L1 | 0.99993 | 0.99981-1.00006 | 0.30917 |
| TFCP2 | 1.00005 | 0.99931-1.00079 | 0.89431 |
| OTX1 | 0.9991 | 0.99814-1.00007 | 0.06955 |
| YEATS4 | 1.00002 | 0.99973-1.00031 | 0.87762 |
| HMGA2 | 1.00004 | 0.99996-1.00013 | 0.28439 |
| HMGA1 | 1.00002 | 0.99998-1.00005 | 0.30922 |
| HAX1 | 1.00033 | 1.00007-1.00059 | 0.01428 |
| NFRKB | 0.99989 | 0.99925-1.00053 | 0.74056 |
| DBP | 0.9996 | 0.99893-1.00028 | 0.24833 |
| MED1 | 0.99998 | 0.99972-1.00024 | 0.88243 |
| TCF4 | 0.99984 | 0.99966-1.00002 | 0.0825 |
| TCF7 | 0.9999 | 0.99927-1.00053 | 0.76273 |
| TCF3 | 1.00013 | 0.99994-1.00031 | 0.17661 |
| PCBD1 | 1.00044 | 1.00009-1.0008 | 0.01495 |
| PITX2 | 1 | 0.99979-1.00021 | 0.99323 |
| PITX1 | 0.99997 | 0.99992-1.00002 | 0.27563 |
| KAT2B | 0.99936 | 0.99881-0.9999 | 0.02111 |
| MAFB | 0.99991 | 0.99983-1 | 0.04502 |
| MAFF | 1.00016 | 0.99986-1.00046 | 0.28428 |
| MAFG | 0.99994 | 0.99961-1.00027 | 0.72824 |
| RBPJ | 1.00001 | 0.9998-1.00023 | 0.91057 |
| TRAF6 | 0.99984 | 0.99859-1.00109 | 0.8016 |
| POLR1A | 0.99985 | 0.99954-1.00015 | 0.3297 |
| NR1H2 | 0.99973 | 0.99943-1.00004 | 0.08475 |
| HOXB7 | 1.00045 | 0.99959-1.00132 | 0.30582 |
| HOXB4 | 0.99766 | 0.99464-1.00069 | 0.12995 |
| SPDEF | 0.9997 | 0.99905-1.00035 | 0.36047 |
| NR2F6 | 1.00026 | 0.99997-1.00056 | 0.08083 |
| NR2F1 | 0.99904 | 0.99761-1.00047 | 0.18782 |
| SNAI2 | 1.00021 | 1.00008-1.00034 | 0.00114 |
| EN1 | 0.99985 | 0.99909-1.00062 | 0.71001 |
| HDAC10 | 1.00089 | 0.99944-1.00235 | 0.22832 |
| TP53 | 0.99996 | 0.99978-1.00013 | 0.63815 |
| TRIB3 | 1.00015 | 1.00002-1.00027 | 0.01943 |
| EZH2 | 1.00026 | 0.9999-1.00062 | 0.15075 |
| NR4A2 | 0.99979 | 0.99927-1.00031 | 0.4256 |
| NR4A3 | 0.9999 | 0.99938-1.00042 | 0.69818 |
| NR4A1 | 1.00002 | 0.99992-1.00011 | 0.74776 |
| NCOA4 | 0.99993 | 0.99984-1.00003 | 0.17993 |
| HIC1 | 1.00041 | 0.99911-1.00172 | 0.53572 |
| RXRA | 0.99991 | 0.99979-1.00002 | 0.11659 |
| RAD51 | 1.00138 | 1.00064-1.00213 | 0.00026 |
| ZNRD1 | 1.0007 | 1.00013-1.00127 | 0.01573 |
| ETV3 | 0.9998 | 0.99943-1.00017 | 0.29727 |
| ETV5 | 1.00008 | 0.99974-1.00042 | 0.65177 |
| ETV4 | 1.00013 | 0.99985-1.00041 | 0.35191 |
| ETV7 | 1.00009 | 0.99958-1.00061 | 0.72178 |
| ETV6 | 0.99959 | 0.99925-0.99993 | 0.01667 |
| IKZF1 | 0.99985 | 0.99929-1.00041 | 0.59981 |
| BRPF1 | 0.99964 | 0.9986-1.00068 | 0.49949 |
| SP1 | 0.99969 | 0.99946-0.99993 | 0.00984 |
| SP2 | 0.99913 | 0.99811-1.00016 | 0.09824 |
| SP3 | 0.99986 | 0.99963-1.00008 | 0.21397 |
| SP4 | 0.99893 | 0.99688-1.00099 | 0.30982 |
| HMG20B | 1.0002 | 1.00002-1.00038 | 0.02698 |
| PBX2 | 1.00032 | 0.99991-1.00073 | 0.12618 |
| PBX1 | 0.99973 | 0.99951-0.99996 | 0.02027 |
| DR1 | 1.00017 | 0.99985-1.0005 | 0.29751 |
| CHD4 | 0.99993 | 0.99982-1.00004 | 0.20985 |
| CHD8 | 0.99981 | 0.99955-1.00007 | 0.15279 |
| ZFP36L1 | 0.99998 | 0.99994-1.00002 | 0.36357 |
| SPEN | 0.99983 | 0.99964-1.00001 | 0.06024 |
| DAXX | 1.00037 | 1.00006-1.00068 | 0.01774 |
| SHOX2 | 1.00028 | 0.99943-1.00112 | 0.52144 |
| HIPK2 | 0.99977 | 0.99956-0.99999 | 0.04249 |
| FOXJ1 | 0.99946 | 0.99827-1.00066 | 0.37836 |
| TCFL5 | 1.0007 | 0.99976-1.00165 | 0.14576 |
| RUVBL1 | 1.00026 | 1.00008-1.00045 | 0.00438 |
| TBC1D22A | 0.99988 | 0.99943-1.00032 | 0.58443 |
| FOXL2 | 1.00112 | 1.00008-1.00217 | 0.03517 |
| BRCA1 | 1.00027 | 0.99969-1.00085 | 0.36224 |
| BRCA2 | 1.00054 | 0.99964-1.00143 | 0.23861 |
| PCGF2 | 1.00064 | 1.00012-1.00116 | 0.01555 |
| NR5A2 | 0.99049 | 0.98172-0.99933 | 0.03504 |
| DLX4 | 0.99949 | 0.9978-1.00119 | 0.55629 |
| HMGN1 | 1.00015 | 1-1.0003 | 0.04817 |
| MDM2 | 0.99988 | 0.99966-1.00009 | 0.25663 |
| MDM4 | 1.00017 | 0.9994-1.00095 | 0.66547 |
| ARNTL2 | 0.99998 | 0.99987-1.0001 | 0.76459 |
| GABPA | 0.99945 | 0.99831-1.00059 | 0.344 |
| GLIS3 | 0.99925 | 0.99825-1.00026 | 0.14453 |
| SREBF2 | 0.99995 | 0.99987-1.00004 | 0.26426 |
| SREBF1 | 0.99995 | 0.99988-1.00002 | 0.16606 |
| MTA1 | 1.0002 | 0.99986-1.00054 | 0.25317 |
| MTA2 | 1.00003 | 0.99987-1.00018 | 0.73863 |
| MTA3 | 1.00077 | 1.00001-1.00152 | 0.0464 |
| BACH2 | 0.9997 | 0.99833-1.00107 | 0.66712 |
| KCNIP3 | 0.99968 | 0.99821-1.00115 | 0.6703 |
| ELK3 | 1.00012 | 0.99993-1.00031 | 0.2186 |
| ELK1 | 1.00012 | 0.99995-1.00028 | 0.16832 |
| ELK4 | 0.99978 | 0.99941-1.00014 | 0.22656 |
| PER2 | 0.99946 | 0.999-0.99992 | 0.02259 |
| KAT5 | 1.00008 | 0.99935-1.0008 | 0.83793 |
| PLAG1 | 0.99963 | 0.9986-1.00067 | 0.4894 |
| JDP2 | 0.99979 | 0.99919-1.00038 | 0.48226 |
| ATF4 | 1.00004 | 0.99998-1.0001 | 0.1588 |
| ATF7 | 0.99909 | 0.9983-0.99989 | 0.02543 |
| ATF6 | 1.00016 | 0.9997-1.00062 | 0.48342 |
| ATF1 | 0.99973 | 0.99853-1.00093 | 0.6617 |
| ATF3 | 1.00004 | 0.99996-1.00013 | 0.324 |
| ATF2 | 1.00002 | 0.99952-1.00052 | 0.92915 |
| LCOR | 0.9998 | 0.99898-1.00062 | 0.62737 |
| TSC22D1 | 0.99997 | 0.99991-1.00004 | 0.44905 |
| TSC22D3 | 1.00002 | 0.99993-1.00012 | 0.61804 |
| TNFAIP3 | 1.00005 | 0.99998-1.00012 | 0.12916 |
| GFI1 | 1.00125 | 0.99978-1.00272 | 0.09486 |
| HINFP | 1.00118 | 0.99972-1.00265 | 0.11301 |
| REL | 0.99947 | 0.99874-1.0002 | 0.15363 |
| ZNF239 | 0.9963 | 0.99078-1.00186 | 0.19141 |
| TAL1 | 0.99787 | 0.99377-1.00198 | 0.30966 |
| FLI1 | 0.99992 | 0.99907-1.00078 | 0.86168 |
| SIRT3 | 1.00001 | 0.99896-1.00105 | 0.99055 |
| SIRT2 | 0.99988 | 0.99964-1.00013 | 0.36171 |
| SIRT1 | 0.99924 | 0.99787-1.00061 | 0.27787 |
| CTNNB1 | 1.00005 | 0.99999-1.0001 | 0.08865 |
| NR2C2 | 0.99955 | 0.99895-1.00015 | 0.13932 |
| MED15 | 1.00003 | 0.99997-1.00008 | 0.34582 |
| APEX1 | 1.00015 | 1.00002-1.00028 | 0.0288 |
| ESRRA | 1.00007 | 0.99979-1.00035 | 0.61634 |
| ESRRB | 0.99045 | 0.98047-1.00052 | 0.06302 |
| BATF | 0.99939 | 0.99791-1.00088 | 0.42202 |
| HEXIM1 | 0.99995 | 0.99948-1.00043 | 0.85139 |
| PML | 1.00002 | 0.99995-1.00009 | 0.57261 |
| DRAP1 | 1.00002 | 0.99995-1.00009 | 0.56477 |
| VEZF1 | 0.99964 | 0.99918-1.00011 | 0.13804 |
| MEF2C | 0.99995 | 0.99982-1.00008 | 0.44491 |
| MEF2B | 0.98767 | 0.96737-1.0084 | 0.24181 |
| MEF2A | 0.99998 | 0.99966-1.0003 | 0.89982 |
| MEF2D | 0.99992 | 0.99964-1.0002 | 0.57058 |
| HIF1A | 1.00001 | 0.99997-1.00005 | 0.70547 |
| PAX6 | 1.00012 | 0.99842-1.00183 | 0.88748 |
| PAX1 | 0.99971 | 0.99935-1.00007 | 0.11494 |
| PAX3 | 0.9994 | 0.99414-1.00469 | 0.82424 |
| PAX8 | 1.00044 | 0.99956-1.00133 | 0.32602 |
| RFXANK | 1.00012 | 0.99994-1.0003 | 0.20217 |
| NCOR1 | 0.99981 | 0.99957-1.00004 | 0.10975 |
| TEF | 0.99963 | 0.99925-1.00001 | 0.0572 |
| LMO2 | 0.99949 | 0.99828-1.00069 | 0.40365 |
| LMO3 | 0.99597 | 0.99018-1.00179 | 0.17421 |
| LMO4 | 0.99976 | 0.99958-0.99994 | 0.00827 |
| UBP1 | 0.99997 | 0.99958-1.00036 | 0.87358 |
| PHB2 | 1.00003 | 0.99995-1.00011 | 0.46839 |
| PKNOX1 | 1.00056 | 0.99922-1.00191 | 0.41134 |
| MCM5 | 1.00005 | 0.99994-1.00016 | 0.35459 |
| MCM2 | 1 | 0.99992-1.00009 | 0.91179 |
| VDR | 1.00013 | 0.99988-1.00037 | 0.30454 |
| NF1 | 0.99987 | 0.99961-1.00013 | 0.3272 |
| KLF10 | 0.99999 | 0.99988-1.0001 | 0.9073 |
| USF2 | 1.00009 | 0.99993-1.00025 | 0.27484 |
| USF1 | 1.0003 | 1.00003-1.00057 | 0.03173 |
| RBMX | 1.00019 | 0.99996-1.00041 | 0.09887 |
| TCF7L2 | 1.00009 | 0.99925-1.00092 | 0.83902 |
| CIITA | 0.99999 | 0.99973-1.00024 | 0.91605 |
| SOX11 | 1.00031 | 0.99961-1.00101 | 0.38328 |
| SOX17 | 0.99718 | 0.99189-1.0025 | 0.29779 |
| HES6 | 0.99987 | 0.99911-1.00062 | 0.72758 |
| MITF | 0.99954 | 0.99867-1.00041 | 0.29877 |
| SUZ12 | 0.99983 | 0.99932-1.00034 | 0.51764 |
| NFE2 | 1.00291 | 0.99889-1.00693 | 0.15623 |
| FOXA1 | 1 | 0.99969-1.0003 | 0.98006 |
| FOXA3 | 1.00035 | 0.9956-1.00512 | 0.88581 |
| FOXA2 | 1.00017 | 0.99508-1.00528 | 0.94847 |
| DACH1 | 0.99339 | 0.9875-0.99932 | 0.02908 |
| TSG101 | 1.00041 | 1.00007-1.00074 | 0.01658 |
| ZEB2 | 0.99986 | 0.99947-1.00026 | 0.49741 |
| SNW1 | 1.00024 | 0.99999-1.00049 | 0.06033 |
| TRIM16 | 1.00008 | 0.9999-1.00026 | 0.35906 |
| SMARCB1 | 1.0002 | 0.99997-1.00043 | 0.0931 |
| PPARGC1A | 0.9994 | 0.99858-1.00023 | 0.15729 |
| JUNB | 1 | 0.99997-1.00004 | 0.88343 |
| JUND | 0.99999 | 0.9999-1.00009 | 0.85499 |
| PPARG | 0.99882 | 0.99706-1.00059 | 0.19238 |
| PPARD | 1.00001 | 0.99989-1.00013 | 0.8931 |
| PPARA | 0.99922 | 0.99868-0.99976 | 0.00443 |
| DDB1 | 0.99997 | 0.99987-1.00006 | 0.48997 |
| DEDD | 1.00067 | 0.99985-1.00148 | 0.10882 |
| IKBKB | 0.9998 | 0.99935-1.00026 | 0.40395 |
| PLAGL2 | 0.99973 | 0.99938-1.00009 | 0.14607 |
| PLAGL1 | 0.99982 | 0.99899-1.00065 | 0.66802 |
| EPAS1 | 0.99995 | 0.99988-1.00002 | 0.17865 |
| E2F7 | 1.00046 | 0.99988-1.00105 | 0.12319 |
| ZNF202 | 1.00076 | 0.99871-1.00282 | 0.46553 |
| ELL | 1.00022 | 0.9994-1.00103 | 0.60311 |
| NAB1 | 1.00011 | 0.99994-1.00029 | 0.18915 |
| NAB2 | 0.99987 | 0.99948-1.00026 | 0.5113 |
| E4F1 | 1.00008 | 0.9994-1.00075 | 0.82388 |
| ESRRG | 0.99799 | 0.99513-1.00087 | 0.17131 |
| ZBTB2 | 1.00027 | 0.99878-1.00177 | 0.71954 |
| ZBTB5 | 0.99957 | 0.99902-1.00012 | 0.12958 |
| TRPS1 | 0.99966 | 0.99931-1.00001 | 0.05709 |
| GRHL1 | 0.99989 | 0.99976-1.00002 | 0.10399 |
| NR3C2 | 0.99707 | 0.99479-0.99935 | 0.01173 |
| NR3C1 | 1.00001 | 0.99973-1.00029 | 0.94408 |
| NFE2L2 | 0.99994 | 0.99989-1 | 0.05126 |
| NFE2L1 | 1.00001 | 0.99998-1.00004 | 0.65858 |
| SCAND1 | 0.99999 | 0.99985-1.00013 | 0.89631 |
| MLLT10 | 1.00017 | 0.99941-1.00093 | 0.65985 |
| SOX9 | 1.00004 | 0.99988-1.0002 | 0.60592 |
| SOX2 | 0.99992 | 0.99983-1.00002 | 0.11301 |
| SOX4 | 0.99995 | 0.99984-1.00006 | 0.35681 |
| SOX5 | 0.99559 | 0.99162-0.99957 | 0.02982 |
| CRTC1 | 0.99836 | 0.99701-0.99971 | 0.01702 |
| BTAF1 | 1.00016 | 0.99963-1.0007 | 0.54332 |
| MSC | 1.00005 | 0.99972-1.00038 | 0.7632 |
| NR1H3 | 1.00044 | 0.99985-1.00102 | 0.14296 |
| SLC2A4RG | 1.00018 | 1-1.00035 | 0.04859 |
| RFXAP | 1.00096 | 0.99624-1.00569 | 0.69189 |
| ELF1 | 0.99992 | 0.99964-1.0002 | 0.57938 |
| ELF3 | 0.99996 | 0.99991-1.00002 | 0.20548 |
| ELF2 | 0.99914 | 0.99814-1.00013 | 0.08913 |
| ELF4 | 0.99993 | 0.9997-1.00016 | 0.53934 |
| AIP | 1.00016 | 0.99997-1.00036 | 0.10506 |
| RNF14 | 1.00045 | 0.99994-1.00096 | 0.08354 |
| FOXQ1 | 0.99987 | 0.9995-1.00025 | 0.51017 |
| HLX | 0.99985 | 0.99809-1.00161 | 0.864 |
| HLF | 0.99945 | 0.99892-0.99999 | 0.0464 |
| SIX1 | 0.9995 | 0.99882-1.00017 | 0.1451 |
| KHDRBS1 | 1.00023 | 0.99998-1.00049 | 0.07385 |
| MYBL1 | 1.0018 | 0.99964-1.00397 | 0.10274 |
| MYBL2 | 1.0001 | 1.00002-1.00019 | 0.02162 |
| SRF | 1.00029 | 1.00002-1.00055 | 0.03299 |
| EIF2AK2 | 1.00006 | 0.99991-1.00021 | 0.40425 |
| HNF4G | 1.00059 | 0.99571-1.00549 | 0.81325 |
| MLX | 1.00071 | 1.00029-1.00113 | 0.00086 |
| DEAF1 | 0.9994 | 0.99876-1.00005 | 0.06972 |
| MECP2 | 0.99989 | 0.99943-1.00036 | 0.64947 |
| GZF1 | 1.00019 | 0.99878-1.00159 | 0.79449 |
| ATM | 0.99973 | 0.99921-1.00026 | 0.32274 |
| TBX21 | 1.00009 | 0.99472-1.00548 | 0.97482 |
| KLF13 | 0.99993 | 0.99982-1.00003 | 0.18262 |
| KLF12 | 0.99984 | 0.99895-1.00074 | 0.73154 |
| KLF11 | 0.99913 | 0.99841-0.99986 | 0.01868 |
| KLF15 | 0.99813 | 0.99525-1.00101 | 0.20223 |
| HHEX | 0.99924 | 0.99731-1.00118 | 0.44485 |
| DNMT3A | 0.9995 | 0.99896-1.00005 | 0.0726 |
| ZNF175 | 0.99859 | 0.9954-1.00179 | 0.38803 |
| NFIL3 | 1.00009 | 0.9998-1.00038 | 0.5512 |
| PTTG1 | 1.00008 | 0.99995-1.00021 | 0.2319 |
| E2F6 | 1.00085 | 0.99881-1.00289 | 0.41598 |
| E2F5 | 1.00138 | 0.9992-1.00356 | 0.21555 |
| E2F4 | 1.00037 | 1.00012-1.00062 | 0.0033 |
| E2F3 | 1.00028 | 0.99946-1.0011 | 0.50332 |
| E2F2 | 1.00024 | 0.99972-1.00077 | 0.35949 |
| E2F1 | 1.00007 | 0.99986-1.00028 | 0.53238 |
| E2F8 | 0.99984 | 0.99858-1.00111 | 0.80911 |
| TBP | 1.00181 | 1.0004-1.00322 | 0.01176 |
| RBBP7 | 1.00011 | 0.99994-1.00028 | 0.21818 |
| MLLT3 | 1.00003 | 0.99919-1.00086 | 0.95206 |
| CDX1 | 1.00474 | 1.00058-1.00892 | 0.02542 |
| TLE3 | 0.99991 | 0.9997-1.00012 | 0.39496 |
| TFPT | 1.00035 | 0.99989-1.00081 | 0.13097 |
| MBD1 | 0.99993 | 0.99972-1.00014 | 0.51367 |
| MBD2 | 1.00009 | 0.99988-1.00031 | 0.38859 |
| IRF3 | 1.00019 | 0.99999-1.00038 | 0.05931 |
| IRF1 | 1.00003 | 0.99994-1.00011 | 0.54376 |
| IRF7 | 1.00007 | 0.99996-1.00019 | 0.20046 |
| IRF6 | 1.00004 | 0.99999-1.00008 | 0.10461 |
| IRF4 | 0.99967 | 0.99884-1.00051 | 0.44685 |
| IRF9 | 1.00175 | 0.99993-1.00357 | 0.05921 |
| IRF8 | 0.99987 | 0.99931-1.00043 | 0.65584 |
| ZBTB17 | 1.00043 | 0.99979-1.00108 | 0.18961 |
| ZBTB16 | 0.99842 | 0.99626-1.00059 | 0.15276 |
| NRF1 | 1.0002 | 0.99814-1.00227 | 0.84821 |
| SERTAD1 | 1.00012 | 0.99984-1.00041 | 0.39444 |
| FOSL1 | 1.00002 | 0.99994-1.0001 | 0.62714 |
| FOSL2 | 0.99991 | 0.99984-0.99998 | 0.01108 |
| MAX | 1.00004 | 0.9997-1.00038 | 0.80841 |
| MAZ | 1.0001 | 0.99972-1.00048 | 0.60523 |
| MAF | 1.00002 | 0.99992-1.00011 | 0.73386 |
| MAL | 0.99999 | 0.99997-1.00001 | 0.23214 |
| IRF2 | 0.99984 | 0.9993-1.00038 | 0.55529 |
| IRF5 | 1.00013 | 0.99952-1.00073 | 0.67706 |
| TBL1X | 0.99967 | 0.99931-1.00003 | 0.07119 |
| SMARCA1 | 0.99994 | 0.9996-1.00029 | 0.73711 |
| SMARCA4 | 0.99982 | 0.99967-0.99996 | 0.01274 |
| PA2G4 | 1.00003 | 0.99995-1.00011 | 0.48555 |
| HOXC9 | 1.00151 | 0.99875-1.00427 | 0.28339 |
| HOXC8 | 0.9998 | 0.99626-1.00334 | 0.91029 |
| HOXC6 | 0.99826 | 0.99531-1.00122 | 0.24958 |
| MSX1 | 1.00005 | 0.99888-1.00122 | 0.93739 |
| MSX2 | 1.00057 | 0.99955-1.00158 | 0.27614 |
| SIN3A | 0.99981 | 0.99943-1.00019 | 0.33459 |
| ZIC2 | 0.99974 | 0.99878-1.00069 | 0.58649 |
| ING4 | 0.99997 | 0.99934-1.0006 | 0.92759 |
| ING1 | 0.99989 | 0.99916-1.00063 | 0.77675 |
| ING2 | 1.00081 | 0.99947-1.00214 | 0.23522 |
| SNIP1 | 0.99985 | 0.99818-1.00151 | 0.85545 |
| NCOR2 | 0.99991 | 0.9998-1.00001 | 0.08574 |
| SUPT3H | 1.0023 | 1.00083-1.00376 | 0.0021 |
| RB1CC1 | 1 | 0.99975-1.00026 | 0.96985 |
| TFEB | 1.00017 | 0.99963-1.0007 | 0.53787 |
| LEF1 | 0.99987 | 0.9992-1.00054 | 0.69848 |
| PGR | 0.99937 | 0.9974-1.00135 | 0.53461 |
| BMI1 | 1.00027 | 0.9996-1.00093 | 0.43402 |
| CBFB | 1.00049 | 1.00006-1.00092 | 0.02584 |
| ZNF24 | 0.99998 | 0.99963-1.00032 | 0.88952 |
| ARNT | 0.99983 | 0.99925-1.00041 | 0.56063 |
| RUNX2 | 1.00005 | 0.99955-1.00055 | 0.83244 |
| RUNX3 | 0.99987 | 0.99959-1.00014 | 0.34001 |
| ESR2 | 0.99996 | 0.99161-1.00838 | 0.99284 |
| ESR1 | 0.99903 | 0.99785-1.00022 | 0.11155 |
| TBX2 | 0.99993 | 0.99946-1.0004 | 0.77578 |
| TBX3 | 0.99997 | 0.99944-1.0005 | 0.91419 |
| BDP1 | 0.99966 | 0.99898-1.00034 | 0.32348 |
| RFWD2 | 1.0002 | 0.99967-1.00073 | 0.46571 |
| AR | 0.99918 | 0.99804-1.00032 | 0.1569 |
| CREB3L4 | 1.00016 | 0.99961-1.00072 | 0.56708 |
| CNOT8 | 1.00058 | 1.00002-1.00113 | 0.04058 |
| CNOT7 | 0.99971 | 0.99932-1.00009 | 0.13726 |
| EAPP | 1.00088 | 1.00035-1.00141 | 0.00119 |
| EGF | 0.99934 | 0.998-1.00069 | 0.34008 |
| GSC | 0.9974 | 0.98777-1.00711 | 0.59826 |
| NFATC3 | 0.99873 | 0.99797-0.99949 | 0.00107 |
| ID4 | 0.99932 | 0.99857-1.00008 | 0.07992 |
| ID2 | 0.99947 | 0.99904-0.99991 | 0.01691 |
| ID3 | 1.00004 | 0.99988-1.0002 | 0.60806 |
| ID1 | 0.99998 | 0.99991-1.00005 | 0.58427 |
| GLI2 | 1.00008 | 0.9999-1.00025 | 0.39158 |
| GLI3 | 1.00001 | 0.99974-1.00027 | 0.95779 |
| GLI1 | 1.00032 | 0.99957-1.00106 | 0.40834 |
| BARX2 | 0.99994 | 0.99976-1.00013 | 0.54979 |
| HIVEP2 | 0.99982 | 0.99952-1.00013 | 0.25245 |
| SFPQ | 1.00005 | 0.99993-1.00016 | 0.42655 |
| FOXK2 | 0.99987 | 0.99954-1.0002 | 0.44498 |
| HCFC1 | 0.9998 | 0.99958-1.00002 | 0.06823 |
| ZNF350 | 1.00094 | 0.99818-1.00371 | 0.50485 |
| TWIST1 | 0.99987 | 0.99934-1.00041 | 0.63928 |
| TWIST2 | 0.99992 | 0.99867-1.00117 | 0.89817 |
| POU2F2 | 1.00011 | 0.9993-1.00093 | 0.78745 |
| POU2F1 | 0.99943 | 0.99852-1.00034 | 0.2213 |
| PRDM1 | 0.99987 | 0.99967-1.00006 | 0.17929 |
| MYC | 1.00001 | 0.99995-1.00008 | 0.71386 |
| MYB | 0.99907 | 0.99793-1.00022 | 0.11215 |
| GATA2 | 1.00029 | 0.99871-1.00187 | 0.72037 |
| GATA3 | 0.99992 | 0.99906-1.00078 | 0.85217 |
| CLOCK | 0.99942 | 0.99889-0.99996 | 0.03441 |
| STAT5A | 0.99993 | 0.99956-1.0003 | 0.70944 |
| ZNF444 | 1.00018 | 0.99952-1.00084 | 0.59369 |
| FOXC2 | 1.00056 | 0.99973-1.00138 | 0.18656 |
| FOXC1 | 0.99986 | 0.99955-1.00018 | 0.39683 |
| MZF1 | 0.99929 | 0.99793-1.00066 | 0.30973 |
| HR | 0.99999 | 0.9999-1.00008 | 0.8459 |
| PROX1 | 0.99934 | 0.99732-1.00135 | 0.51853 |
| GTF2B | 1.00075 | 0.99999-1.0015 | 0.05204 |
| GTF2I | 0.99945 | 0.99908-0.99982 | 0.00355 |
| CREBBP | 0.99968 | 0.99948-0.99988 | 0.00182 |
| HEYL | 1.0002 | 0.9995-1.0009 | 0.57017 |
| RBL1 | 1.00042 | 0.99975-1.00109 | 0.21559 |
| RBL2 | 0.99984 | 0.99949-1.00019 | 0.38244 |
| HEY2 | 0.9983 | 0.99559-1.00101 | 0.21766 |
| HEY1 | 1.00001 | 0.99978-1.00025 | 0.90907 |
| MTF1 | 0.99927 | 0.99871-0.99984 | 0.01175 |
| ZNF224 | 1.00116 | 0.99883-1.0035 | 0.32821 |
| HOXC11 | 1.00299 | 1.00115-1.00483 | 0.00144 |
| HOXC10 | 1.00049 | 0.99956-1.00142 | 0.30234 |
| HOXC13 | 1.00095 | 1.00015-1.00175 | 0.02013 |
| SPI1 | 1.00009 | 0.99967-1.00051 | 0.66839 |
| SPIB | 0.99997 | 0.99981-1.00013 | 0.71221 |
| SIM2 | 0.99966 | 0.9992-1.00011 | 0.13486 |
| LDB1 | 1.00006 | 0.99969-1.00043 | 0.73244 |
| SATB2 | 1.00021 | 0.99866-1.00177 | 0.78703 |
| SATB1 | 0.99879 | 0.99803-0.99954 | 0.00162 |
| PHF10 | 0.9999 | 0.99946-1.00035 | 0.67425 |
| BCL11A | 0.99927 | 0.99873-0.99981 | 0.00842 |
| POU2F3 | 0.99709 | 0.99468-0.99951 | 0.01862 |
| ARID1B | 0.9995 | 0.99921-0.9998 | 0.00092 |
| ARID1A | 0.99979 | 0.99958-0.99999 | 0.03568 |
| MKL1 | 1 | 0.99969-1.00031 | 0.99855 |
| NHLH2 | 0.99933 | 0.99778-1.00089 | 0.40242 |
| SND1 | 0.99997 | 0.99986-1.00008 | 0.59328 |
| TGIF1 | 1 | 0.99987-1.00013 | 0.98947 |
| STAT6 | 0.99993 | 0.99981-1.00006 | 0.30835 |
| STAT4 | 1.00018 | 1-1.00035 | 0.04433 |
| STAT3 | 0.99988 | 0.99981-0.99996 | 0.00302 |
| STAT2 | 1.00006 | 0.99997-1.00014 | 0.17184 |
| STAT1 | 1.00001 | 0.99999-1.00003 | 0.2116 |
| ZNF76 | 1.00047 | 0.99961-1.00134 | 0.28466 |
| NFIC | 0.99984 | 0.9997-0.99997 | 0.01956 |
| NFIB | 0.99977 | 0.99956-0.99999 | 0.03818 |
| NFIA | 0.9992 | 0.99872-0.99967 | 0.00093 |
| NFIX | 0.99984 | 0.99969-0.99998 | 0.02805 |
